# Mitochondrial DNA depletion by ethidium bromide decreases neuronal mitochondrial creatine kinase: Implications for striatal energy metabolism

**DOI:** 10.1371/journal.pone.0190456

**Published:** 2017-12-29

**Authors:** Emily Booth Warren, Aidan Edward Aicher, Joshua Patrick Fessel, Christine Konradi

**Affiliations:** 1 Department of Pharmacology, Vanderbilt University, Nashville, Tennessee, United States of America; 2 Department of Biological Sciences, Vanderbilt University, Nashville, Tennessee, United States of America; 3 Department of Cancer Biology, Vanderbilt University, Nashville, Tennessee, United States of America; 4 Department of Medicine, Division of Allergy, Pulmonary, and Critical Care Medicine, Vanderbilt University School of Medicine, Nashville, Tennessee, United States of America; 5 Department of Psychiatry, Vanderbilt University, Nashville, Tennessee, United States of America; 6 Vanderbilt Brain Institute, Vanderbilt University, Nashville, Tennessee, United States of America; 7 Kennedy Center for Research on Human Development, Vanderbilt University, Nashville, Tennessee, United States of America; Rutgers University, UNITED STATES

## Abstract

Mitochondrial DNA (mtDNA), the discrete genome which encodes subunits of the mitochondrial respiratory chain, is present at highly variable copy numbers across cell types. Though severe mtDNA depletion dramatically reduces mitochondrial function, the impact of tissue-specific mtDNA reduction remains debated. Previously, our lab identified reduced mtDNA quantity in the putamen of Parkinson’s Disease (PD) patients who had developed L-DOPA Induced Dyskinesia (LID), compared to PD patients who had not developed LID and healthy subjects. Here, we present the consequences of mtDNA depletion by ethidium bromide (EtBr) treatment on the bioenergetic function of primary cultured neurons, astrocytes and neuron-enriched cocultures from rat striatum. We report that EtBr inhibition of mtDNA replication and transcription consistently reduces mitochondrial oxygen consumption, and that neurons are significantly more sensitive to EtBr than astrocytes. EtBr also increases glycolytic activity in astrocytes, whereas in neurons it reduces the expression of mitochondrial creatine kinase mRNA and levels of phosphocreatine. Further, we show that mitochondrial creatine kinase mRNA is similarly downregulated in dyskinetic PD patients, compared to both non-dyskinetic PD patients and healthy subjects. Our data support a hypothesis that reduced striatal mtDNA contributes to energetic dysregulation in the dyskinetic striatum by destabilizing the energy buffering system of the phosphocreatine/creatine shuttle.

## Introduction

Highly energetic tissues such as heart, skeletal muscle, and brain are dependent on efficient energy metabolism for proper function. The two principal cell types of the brain, neurons and astrocytes, are metabolically coupled on multiple levels, through the exchange of oxidizable substrates, anaplerotic intermediates, and amino acid neurotransmitters [1–4]. Interestingly, these two cell types have distinct metabolic phenotypes, as astrocytes increase glycolytic activity in response to activity-dependent demands [5,6], while neurons are unable to upregulate glycolysis [7] and are consequently more dependent on mitochondrial function for the generation of ATP.

Neurons and astrocytes express different subtypes of creatine kinase, the enzyme catalyzing the phosphorylation of creatine (Cr) and dephosphorylation of phosphocreatine (PCr). Creatine is a small organic acid that, as PCr, transports phosphates from sites of ATP synthesis (e.g., the mitochondrial cristae) to sites of ATP consumption [8,9]. Most cell types express a mitochondrial and a cytosolic creatine kinase. However, in the brain, neurons express predominantly the mitochondrial isoform (mtCK), while astrocytes express predominantly the cytosolic isoform (B-CK) [10,11]. Though the functional significance of this enzymatic segregation is currently unknown, it suggests a differential utilization of creatine between the two cell types.

**Fig 1:**
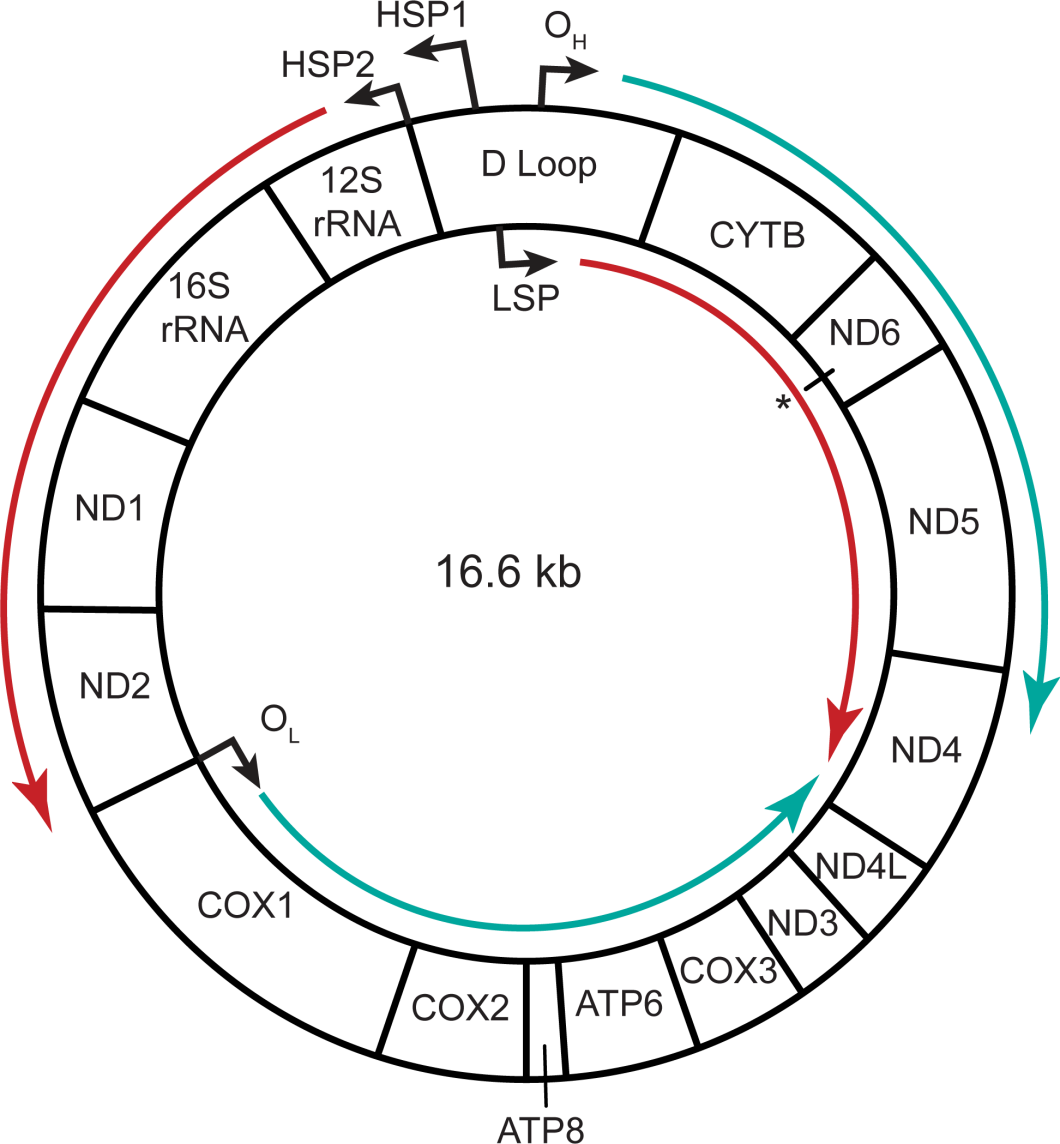
The mitochondrial genome (mtDNA). rRNA-encoding and protein-encoding genes are shown; tRNA-encoding genes are withheld for clarity. Teal arrows depict the direction of mtDNA replication from the heavy strand or light strand origins of replication (O_H_, O_L_); red arrows depict the direction of polycistronic transcription from the heavy strand or light strand promoters (HSP1/2, LSP). *—ND6 is the only protein-encoding gene on the light chain. Adapted from [29].

Despite their metabolic differences, both neurons and astrocytes depend on mitochondrial oxidative phosphorylation [12–15]. The mitochondrial proteome contains over 1500 proteins encoded in nuclear DNA [16] (including mtCK), but mitochondrial function also hinges on the integrity and quantity of the discrete mitochondrial genome (mtDNA), (Fig 1). mtDNA encodes 13 proteins, as well as 22 tRNAs and 2 rRNAs, which are essential subunits of the mitochondrial respiratory chain, which generates ATP by oxidizing NADH and FADH_2_ [17,18]. Cells contain between 10^3^−10^4^ copies of mitochondrial DNA [19,20], while each individual mitochondrion may contain between 1–10 mtDNA plasmids [21,22]. mtDNA copy number ranges across tissue types and across brain regions [23]. Copy number is predominantly determined by the energy requirements of a given tissue [24]. It is in part regulated by methylation of the mtDNA polymerase, *PolgA* [25,26], though expression of the primary mtDNA transcription factor, *Tfam*, can also alter mtDNA quantity [27,28].

Reduction in mtDNA copy number outside the CNS is frequently associated with aging [30–32], though a recent study has called into question the strength of this relationship in the brain [33]. Decreased brain mtDNA copy number has been more consistently associated with various neurological conditions, including Parkinson’s Disease (PD) [34–36], Alzheimer’s Disease [33,37,38], and psychiatric disorders [39,40]. Despite these observations, functional characterizations of the effect of reduced mtDNA quantity on mitochondrial respiratory or bioenergetic capacity have not been definitive. While the pathophysiology of mtDNA depletion syndromes highlights the detrimental effects of insufficient mtDNA copy number [41,42], the threshold of mtDNA reduction across different tissues that will result in reduced mitochondrial function remains unclear.

Previously, our lab reported that PD patients with L-DOPA Induced Dyskinesia (LID) had lower levels of mtDNA in the putamen relative to non-dyskinetic PD patients and healthy controls, but the study could not establish if this was cause or effect [43]. LID is a frequently observed motor disorder of painful, uncontrolled movements that results from L-DOPA therapy of PD [44], with a highly variable individual time course of progression [45,46]. Mitochondrial pathology is a central theme in PD itself [47–49], and a recent animal study has demonstrated that reintroducing healthy mitochondria may improve parkinsonian symptoms [50]. Thus, deficiencies in mitochondrial function might cause PD by degrading neurons in the substantia nigra, and cause LID in the striatum in response to treatment.

Creatine has been used as an experimental therapeutic to improve bioenergetic function and delay neurodegeneration, albeit more successfully in animal models [51,52] than in clinical trials [53,54]. The relevance of mitochondrial dysfunction and creatine homeostasis to the development of LID has not been explored to date.

In the present study, we examined the metabolic consequences of reduced mtDNA levels in rat striatal neuron-enriched co-cultures (NECos), striatal neurons, and astrocytes using EtBr exposure. We utilized low concentration exposures of EtBr to selectively reduce mtDNA without affecting nuclear DNA, similar to previous reports [55]. EtBr causes mtDNA depletion in a rapid, reproducible, and dose-dependent manner [56–58]. NECos were used as the primary model to maintain the metabolic coupling between neurons and glia [59,60], while we used pure neuronal and astrocytic cultures to identify cell type-specific responses. We hypothesized that these two cell types may have different bioenergetic responses to reduced mtDNA quantity due to their differential metabolic phenotypes. We observed that reduced mtDNA reduced mitochondrial respiration in both cell types, but selectively increased astrocyte glycolytic activity, and reduced neuronal expression of mtCK and production of phosphocreatine. Additionally, we confirmed that mtCK is lower expressed in the dyskinetic PD patient cohort with reduced mtDNA. Collectively, our findings provide insight into the bioenergetic repercussions of decreased mtDNA on the different cell types of the striatum, which may contribute to the variability in susceptibility to LID progression.

## Methods

### Primary rat striatal cultures

All animal experiments were conducted under institutional guidelines and were approved by the Institutional Animal Care and Use Committee of Vanderbilt University. Striata (for neuronal cultures and neuron-enriched cocultures (NECos) or cortices (for astrocyte cultures) were dissected from E18 Sprague-Dawley rat fetuses (Charles River Laboratories, Raleigh, NC), as described previously (NECo and neuronal cultures, see [61]; astrocyte cultures, see [62]) with minor modifications. Pregnant dams were anesthetized/euthanized with an overdose of pentobarbital, 130mg/kg. During anesthesia, E18 fetuses were removed, and the diaphragm of dams was cut. After mechanical dissociation in HBSS/5mM HEPES, cells were plated in medium containing 2.5 mM glucose, which approximates physiological CNS glucose concentrations [63,64]. Media glucose concentration was assayed daily, using a glucose oxidase-dependent assay (as described in [65]) Reagents for the glucose assay (glucose oxidase, horseradish peroxidase, and ABTS) were purchased from Sigma-Aldrich (St. Louis, MO). Media glucose was daily restored to 2.5 mM, and cell viability was carefully monitored. Representative traces of media glucose concentrations for each culture condition are shown in S1 Fig. GFAP (1:750, Abcam, Cambridge, MA) immunostaining showed that astrocyte cultures were >90% astrocytic, and ß3-tubulin (1:1000, Cell Signaling Technologies, Cambridge, MA) immunostaining confirmed that neuronal cultures were >95% pure. NECos were approximately 75% neuronal and 25% astrocytic. A minimum of two dissections was performed for each experiment, unless otherwise noted. After plating, all wells or plates were considered biological replicates. No normalization to dissection was performed.

NECos and neuronal cultures were grown in 3:1 DMEM:F12 supplemented with 1 x B27, penicillin (100 U/ml)/streptomycin (100 μg/ml), 2.5 mM pyruvate, and 2.5 mM glucose. NECos were seeded at 1.3*10^5^ cells/cm^2^, and at 3.8*10^5^ cells/cm^2^ for Seahorse experiments. Neurons were seeded at 1.6*10^5^ cells/cm^2^ and at 4.7*10^5^ cells/cm^2^ for Seahorse experiments. To inhibit astrocyte growth in neuronal cultures, 5 μM AraC was added on DIV2 for 24 hours. Experiments with NECos and neurons concluded on DIV7.

Astrocyte media contained 10% FBS instead of B27 to promote astrocyte proliferation. Cells were plated at 1.3*10^5^ cells/cm^2^ and grown to confluence (DIV6-10). Media was replaced every two days. Once confluent, astrocytes were re-seeded at 3.25*10^4^ cells/cm^2^ and at 9.4*10^4^ cells/cm^2^ for Seahorse experiments. Astrocytes were assayed 5 days after re-seeding. EtBr (1% solution, 10 mg/mL) was added to cultures as indicated in each experiment. All cell culture materials were purchased from Thermo Fisher Scientific (Waltham, MA). Creatine for neuronal supplementation was purchased from Sigma.

### Post mortem samples

Human putamen samples were collected at the Harvard Brain Tissue Resource Center at McLean Hospital, Harvard Medical School, Belmont, MA (HBTRC; http://www.brainbank.mclean.org), as previously described [43]. Analysis was carried out in the putamen of 12 control individuals, 10 individuals with PD and LID, and 10 individuals with PD without LID, all of whom were male (demographic information in S1 Table).

All medical records were carefully examined for any symptoms attributable to LID, under observation of all HIPAA and IRB guidelines. Medical records were scanned for clinical symptoms such as ‘dyskinesia’, ‘chorea’, ‘wearing off’, ‘on-off periods’ and prescription of Comtan (entacapone) or Tasmar (tolcapone), and recorded together with information on medications, signs of PD, L-DOPA prescription records, neuropathological findings, signs of dementia and cause of death. From the prescription records the total amount of L-DOPA administered over the entire treatment period divided by the prescription years is given as L-DOPA exposure per year. Exclusion criteria included incomplete medical records, exposure to environmental toxins and use of respiratory devices before death. Investigators were blinded to the diagnosis [43].

### Determination of mtDNA, mtRNA, and mRNA levels

DNA was extracted using the DNeasy kit (Qiagen, Valencia, CA). qPCR analysis was carried out with 20 ng of DNA with SYBR green I for detection of product. For culture experiments, three mitochondrial DNA (mtDNA) primer pairs were used for mtDNA analysis, and three nuclear DNA (nDNA) primer pairs for reference genes; for human experiments, six mitochondrial primer pairs and two reference genes were used, as previously described [43]. As no difference was observed in the levels or pattern of the mtDNA primer pair products, they were combined for the analysis and normalized to the nDNA primer pair products as described [66].

RNA was extracted from human tissue samples with the RNeasy kit (Qiagen) after rotor-stator homogenization. Complementary DNA (cDNA) synthesis was carried out with 150 ng total RNA using the SuperScript IV cDNA synthesis kit and random hexamers (Thermo Fisher Scientific). Rat samples were harvested in RNeasy lysis buffer and extracted according to protocol. 150 ng of total RNA was used in the iScript cDNA synthesis kit (Bio-Rad, Hercules, CA). The KAPA SYBR FAST master mix (KAPA Biosystems, Wilmington, MA) was used for the PCR reaction, with ROX loading control.

All qPCR reactions were performed using a Strategene MX3000 or MX3005p (Agilent, Santa Clara, CA), and a meltcurve analysis from 55°C to 90°C was performed after the last PCR cycle. Standard curves with four 1:4 serial dilutions were run for each primer pair to determine its efficiency. The highest and lowest dilutions of the standard curve plus a non-template control and a no reverse transcriptase control were examined on a 3% agarose gel to verify product size and primer specificity. A minimum of two reference genes was used for normalization of RNA measurements. These genes were selected as stable references following BestKeeper analysis [67]. Efficiency-corrected log2 fold changes were calculated as described [66]. See S2 Table for primer sequences and efficiencies.

### RNASeq

mRNA samples, prepared as above, were used in the TruSeq stranded mRNA reagents kit (Illumina) and paired-end sequenced (PE75) on a HiSeq3000 (Illumina) in the VANTAGE core at Vanderbilt University. Three samples were analyzed per group, each pooled from two independent dissections. The MultiRankSeq package was used for expression comparisons between groups (https://github.com/slzhao/MultiRankSeq), which uses a multimethod rank-sum approach for RNAseq expression analysis that includes DESeq2, edgeR, and baySeq [68]. We present data ranked in DESeq2, with log2-fold change between groups, and false discovery rate–adjusted p-values, though no differences in outcome were observed with data from edgeR or baySeq. All samples passed quality control criteria. Cluster analyses of genes in the top 5% of coefficient of variation are shown in S2 Fig.

For gene network and pathway analysis, we used DAVID bioinformatics resources v6.8 [69]. To avoid excessive duplication across the many available databases, we focused on the UniProt (http://www.uniprot.org/) and KEGG databases (http://www.genome.jp/kegg/). The 500 strongest downregulated and 500 strongest upregulated genes (ranked with DESeq2), together making up ~ 5% of all genes detected, were used for the analysis.

### Extracellular metabolic flux analyses

The Seahorse XFe96 bioanalyzer was used to conduct all experiments (Agilent). One hour before the start of the assay, media was replaced with XF Base Media (Agilent) supplemented with 2.5 mM glucose, 2 mM glutamine, and 1 mM pyruvate. The MitoStress Test program was used for recording three mix and measure cycles following each drug addition. 1 μM of oligomycin, FCCP, rotenone and antimycin were determined to be the most effective concentrations in an independent experiment. Following the experiments, cells were fixed with 10% formalin and Hoechst (1:2000, Thermo Fisher Scientific) stained for cell counting. Cells were counted using the MetaXpress imager (Molecular Devices, Sunnyvale, CA) with automated software. Each OCR measurement was normalized to cell number.

### NADH measures

Relative NADH was quantified using an MTS assay (Cell Titer 96 Aqueous One Solution Cell Proliferation Assay, Promega, Madison, WI), which measures the transfer of electrons to a tetrazolium compound. Assays were incubated for an average of 2 hours. After the assay was completed, cells were fixed, Hoechst stained, and counted as described above. The MTS assay data were comparable to data from a NAD+/NADH Assay (NADH-Glo, Promega) (S3 Fig).

### ADP/ATP measures

Relative ADP and ATP quantities were determined with an ADP/ATP assay (Biochain, Newark, CA) and data normalized to average cell number per treatment per experiment.

### ^1^H NMR spectroscopy

Samples were prepared as described [70] with minor modifications. Cultures were grown in 100 mm plates, rinsed 3 times with ice-cold saline, and harvested in 4 mL MeOH at -80°C. Samples were homogenized with an ultrasonicator, and 4 mL ice cold chloroform and 4 mL ice cold H_2_O were added. The samples were vortexed for 30 seconds and centrifuged for 1 hour at 3600 rpm and 4°C. The supernatant was transferred to a new tube with a small amount Chelex-100 and Fluka pH indicator (Sigma-Aldrich), vortexed for 30 seconds, and centrifuged for 15 minutes at 3600 rpm and 4°C. The supernatant was vacuufuged and reconstituted in 200 μL 5 mM Na_(2)_H_(2)_PO_4_ at pH 7.0 (Sigma-Aldrich) in D_2_O (99.9%, Cambridge Isotope Labs, Andover, MA) containing 0.25 mM sodium trimethylsilyl propionate-d4 (TMSP, 98%, Cambridge Isotope Labs). Sample pH was re-adjusted, and transferred to a 3 mm NMR tube (Bruker BioSpin, Rheinstetten, Germany).

^1^H NMR spectra were recorded using a Bruker AVANCE III 600 MHz spectrometer equipped with a 5 mm CPQCI cryoprobe (Bruker), at 298K. A water signal presaturation sequence (zgpr) was used, with the following acquisition parameters: 256 scans, 64k data points, 14 ppm spectral width, 4s acquisition time, 6s relaxation delay. An independent inversion-relaxation T1 experiment was performed using Cr, PCr, ATP, ADP, and TMSP standards to calculate an appropriate relaxation delay. For PCr, we calculated a T1 relaxation of 1.3s at the 3.0 ppm peak. For Cr, T1 was 1.9s at the 3.0 ppm peak. ATP and ADP overlap in the ^1^H spectrum, and the T1 at their 8.54 ppm peak was 316ms. 6s was determined to be a sufficient relaxation delay for these compounds. The relaxation time of TMSP was 3.27s. Because the difference in amplitude between a 20s and 6s relaxation was 6%, this was applied as a scaling factor to concentration calculations.

Spectra were processed using Topspin software (v3.5, Bruker) with automatic apodization and line broadening (0.3Hz), and manual reference, phase, and baseline correction. Peaks were identified and assigned using Chenomix (v8.2) and cross-referenced against the Human Metabolomic Database (hmdb.ca). Peak fitting and deconvolution were performed using ACD/SpecManager (v12, Advanced Chemistry Development, Toronto, Canada). Concentrations were calculated using peak areas by reference to TMSP. Cell number was extrapolated from seeding density and aggregated cell counting data from similar experiments. Final metabolite concentrations are expressed as nmol/10^7^ cells.

### Statistical analysis

For all experiments with two groups, means were compared with the unpaired two-tailed Student’s *t* test. Within each experiment, p values were corrected for multiple comparisons using the Benjamini-Hochberg correction [54]. For all experiments with more than two groups, ANOVA or Kruskal-Wallis tests were performed (based on evenness of sample number between groups), followed by Tukey HSD or Dunn’s post hoc tests, respectively. All error bars reflect SEM. For qPCR experiments, SEM of both control and treated groups are combined using the delta method as described [66]. All statistical analyses were conducted in R (cran.r-project.org). A difference in mean was considered statistically significant when p < 0.05.

## Results

### EtBr dose- and time-dependently reduces mtDNA in rat primary cultures

**Fig 2:**
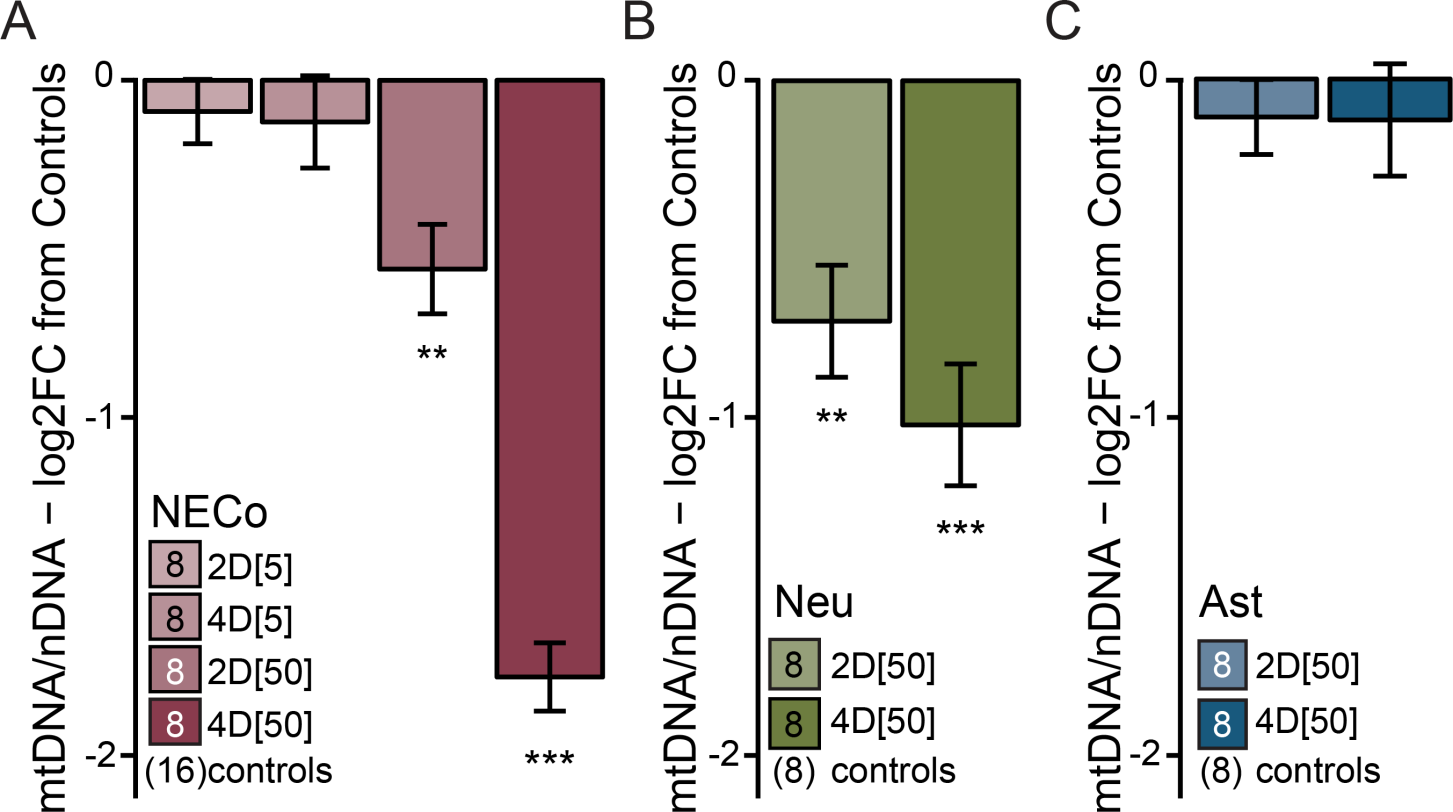
Exposure to EtBr dose- and time-dependently decreases mtDNA in rat striatal co-cultures and purified neuronal cultures, but not in astrocytes. (A-C) Log2-fold change in nDNA-normalized mtDNA quantity in response to EtBr treatment, in (A) neuron-enriched cocultures (NECo), (B) pure neuronal cultures (Neu), and (C) astrocytes (Ast). N for each group is included in figure legends. *** = p < 0.001, ** = p < 0.01, relative to vehicle-treated controls following Dunn’s post-hoc test.

EtBr doses and exposure durations were optimized in NECos. Treatment with 50 ng/ml EtBr two or four days before harvest (2D[50] and 4D[50], respectively) significantly reduced the relative quantity of mtDNA compared to controls. In contrast, 5 ng/ml EtBr applied for two or four days (2D[5], 4D[5]) did not reduce mtDNA (Kruskal Wallis (KW) chi = 28.97, p<0.0001) (Fig 2A). The most effective concentrations (2D[50] and 4D[50]) were repeated in pure neuron and astrocyte cultures, where a similar response was observed in neurons (F = 18.57, p<0.0001), (Fig 2B), but not in astrocytes (Fig 2C). However, astrocyte mtDNA was not entirely resistant to EtBr treatment, as mtDNA and mtDNA-encoded RNA (mtRNA) were significantly reduced by treatment with 500 ng/mL EtBr over 4 days (S4 Fig). In all experiments cultures were maintained at 2.5 mM glucose to mimic brain levels (S1 Fig).

**Fig 3:**
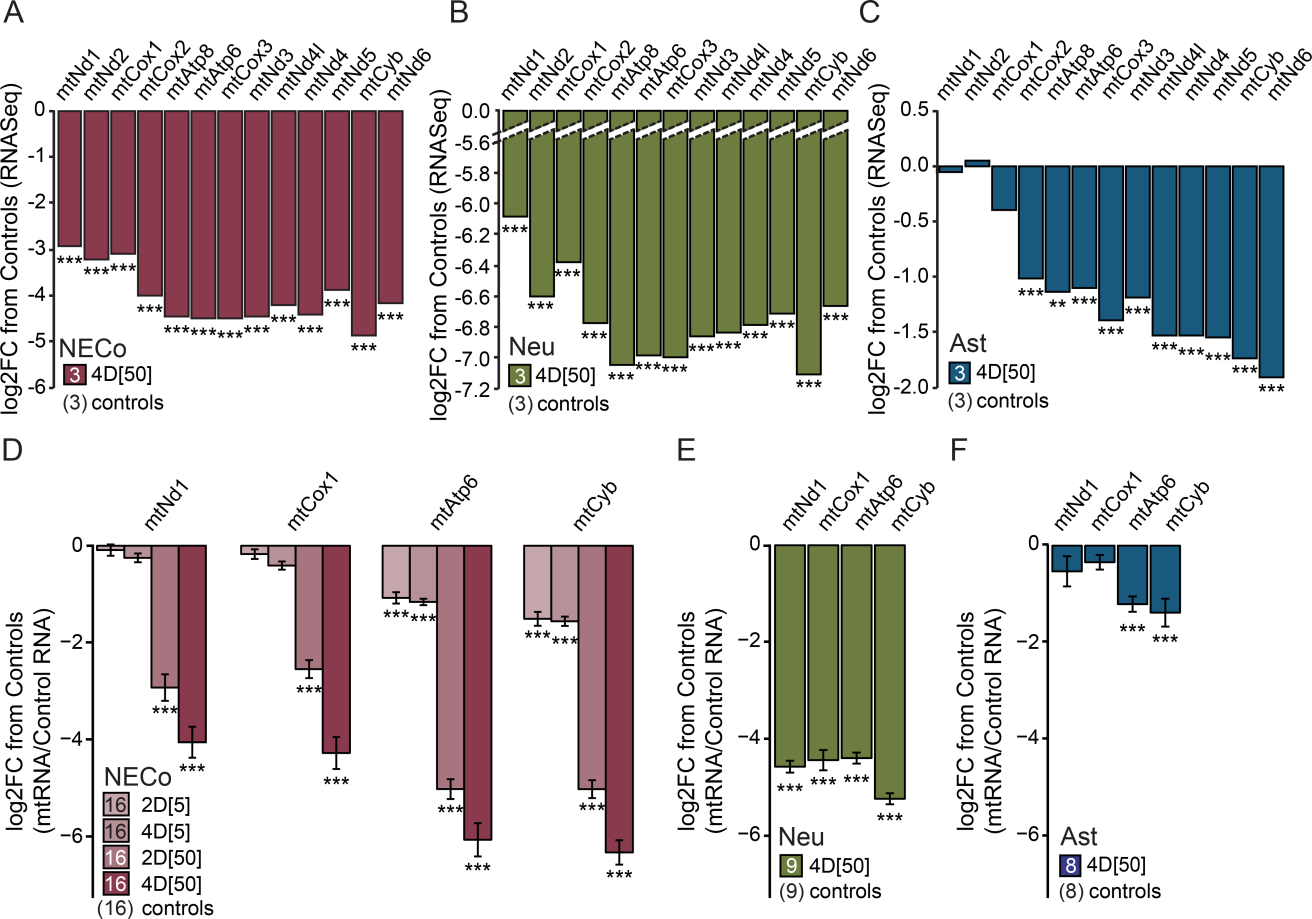
Exposure to EtBr decreases mtRNA in rat striatal co-cultures, purified neuronal cultures, and astrocytes. (A-C) Log2-fold decrease in mtRNA expression after EtBr treatment assayed with RNASeq, in (A) NECos, (B), pure neuronal cultures, and (C) astrocytes. Each bar is a comparison of three control samples to three EtBr-treated samples, each pooled from two independently dissected culture experiments. MultiRankSeq analysis with false discovery rate–adjusted p-values taken from the DESeq comparison. mtRNAs were the most significantly changed RNA transcripts in neurons with a p-value of 0. (D-F) Log2-fold decrease in mtRNA expression after EtBr treatment assayed with qPCR. Error bars reflect delta-method propagated +/-SEM, with level of significance determined following Dunn’s post-hoc test. 2D[5] = 5ng/ml EtBr for 2 days, 4D[5] = 5ng/ml EtBr for 4 days, 2D[50] = 50ng/ml EtBr for 2 days, 4D[50] = 50ng/ml EtBr for 4 days. N for each group is included in figure legends. *** = p < 0.001, ** = p < 0.01, relative to controls. Transcripts are arranged according to their distance from HSP2 (see Fig 1A). The ND6 gene, the only mRNA on the light chain, is shown last.

The effect of EtBr treatment on RNA expression was examined in RNASeq experiments, with the dual purpose of quantifying the effect of EtBr on mtDNA-encoded genes, and to examine gene pathways affected by EtBr. RNASeq experiments were performed after treatment with 50 ng/ml EtBr for four days before harvest (4D[50]), and we observed a significant downregulation in mtRNAs in all three cell types (Fig 3A–3C). mtRNAs made up the top group of downregulated genes in neurons, and mtRNAs in NECos were similarly robustly downregulated. mtRNAs were also significantly downregulated in astrocytes, though the magnitude of the reduction in expression was lesser than that observed in neurons and NECos. After ranking genes according to their coefficients of variation, we determined that genes ranked in the top 5% correctly clustered EtBr-treated samples from controls in all cell types, as shown by heatmaps (S2 Fig). Among the 20 genes with the strongest differential expression by EtBr in neuronal cultures were 14 mtDNA-encoded genes (S3 Table). Although these 20 genes were all downregulated, the overall percentage of downregulated and upregulated genes was equal (S4 Table), indicating that the downregulation of mtRNAs was not due to a downregulation of the entire transcriptome.

The downregulation of four mtRNAs (mtNd1, mtCox1, mtAtp6, mtCytb) after EtBr treatment was confirmed with qPCR in of NECos (Fig 3D), (mtNd1 –F = 125.5, p<0.0001; mtCox1 –F = 150.2, p<0.0001; mtAtp6 –F = 298.1, p<0.0001; mtCytb–F = 421.1, p<0.0001), neurons (Fig 3E), and glia (Fig 3F), with extended dose- and time analyses in NECos. Interestingly, the pattern of mtRNA downregulation reflected the distance of each gene from the promoter, with genes further away showing greater downregulation (see Fig 1).

For a discovery-driven analysis of RNASeq data we used NIH DAVID, with focus on the UniProt and KEGG databases, which have in-depth characterization of gene groups. The 500 most significantly downregulated and 500 most significantly upregulated genes, ranked with DESeq2, were used in separate analyses (S5 Table). The combined group of these genes made up less than 5% of all genes analyzed. The results with these unbiased groups of genes that were selected solely on their different expression levels between treatments, mirrored the findings in Fig 3. All cultures showed a downregulation of mitochondrial pathways, with the strongest effect in neurons and NECos, and a more modest effect in astrocytes. Glycolysis pathways were upregulated in astrocytes and NECos, but not in neurons. Furthermore, all cell types exhibited a relative upregulation of genes involved in amino acid metabolism pathways, indicative of adaptations in the glutamate-glutamine-GABA cycle between neurons and astrocytes [59], and an upregulation of gene transcription and translation machinery.

Since mtRNAs were consistently downregulated with 50 ng/ml EtBr in astrocytes, we proceeded to directly compare the three culture conditions under the 4D[50] dose for all subsequent experiments.

### mtDNA and mtRNA reduction decrease mitochondrial oxygen consumption

**Fig 4:**
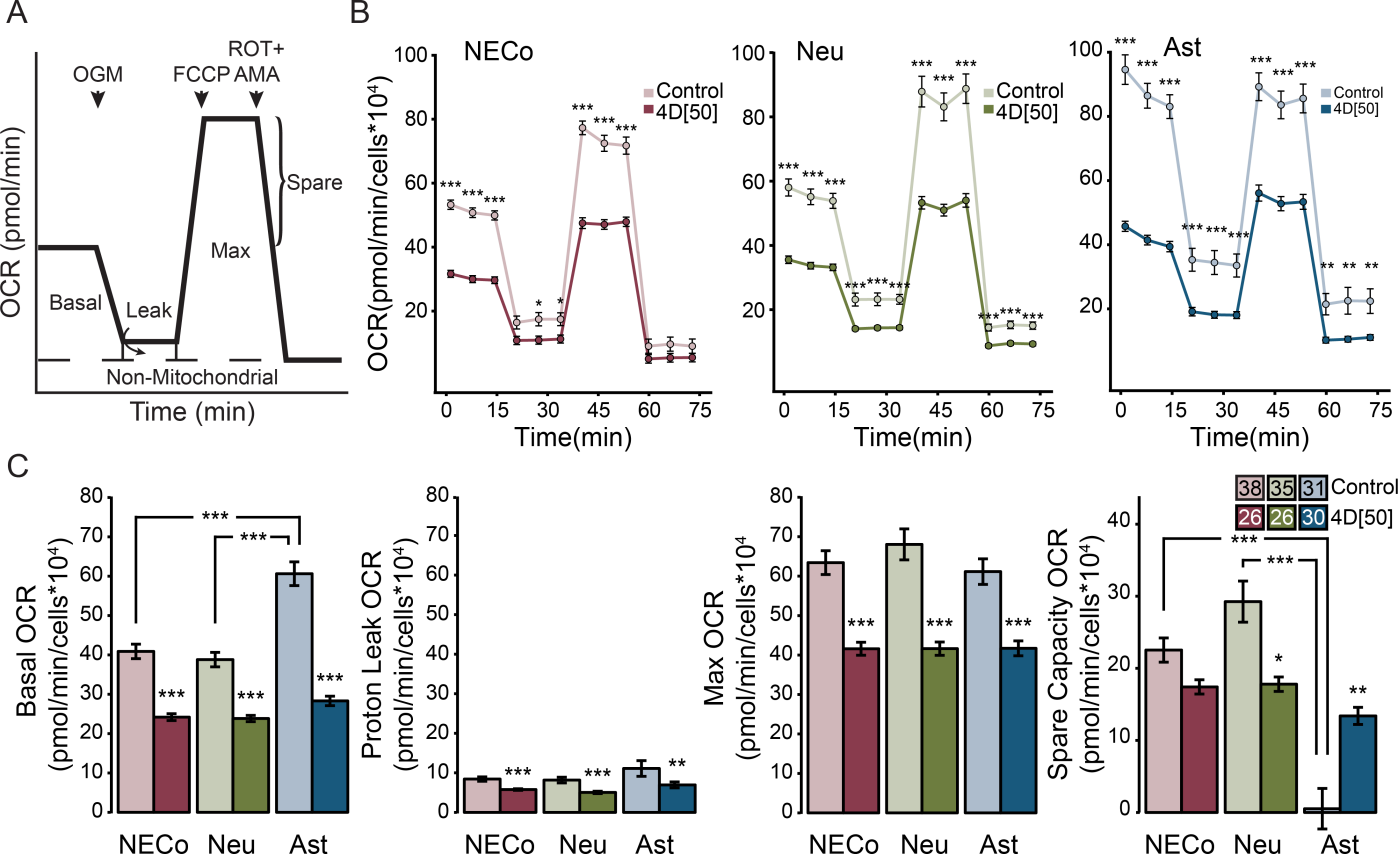
EtBr treatment reduces oxygen consumption and increases mitochondrial spare capacity in astrocytes. (A) Diagram of the procedure and measurements of the Seahorse assay. The sequential addition of mitochondrial toxins permits the measurement of different respiratory states. After recording basal respiration, oligomycin (OGM) is added to block complex V and to eliminate ATP production-linked oxygen consumption. Addition of FCCP allows the free flux of protons through the mitochondrial inner membrane and maximum oxygen consumption. Rotenone and antimycin A (ROT+AMA) inhibit complex I and III, and prevent proton pumping. Non-mitochondrial residual oxygen consumption is subtracted from all measurements. (B) Oxygen consumption rate (OCR) of NECos, neurons, and glia, in the presence and absence of EtBr. (C) Basal, leak, max, and spare capacity OCR of NECos, neurons, and astrocytes, in the presence and absence of EtBr. N for each group is included in figure legends. Error bars reflect +/-SEM. *** = p < 0.001; ** = p < 0.01; * = p < 0.05, after Dunn’s post-hoc test.

To examine the functional consequences of mtDNA and mtRNA reduction, we measured oxygen consumption rate (OCR) in the Seahorse extracellular flux analyzer (Fig 4A). In NECo, neuron, and astrocyte preparations, a reduction in OCR was observed in all mitochondria-linked stages (Fig 4B). EtBr significantly reduced basal, maximum, and leak OCR (basal–KW chi = 120.8, p<0.0001, leak–KW chi = 66.82, p<0.001, max–KW chi = 79.9, p<0.0001) (Fig 4C). Comparing across the three culture conditions in the untreated state shows that, while the maximum OCR of all cultures was similar, the most dramatic difference was between the basal OCR of astrocytes and neurons. Astrocyte basal OCR was not different from its maximum OCR, indicating a lack of spare capacity, whereas neuronal basal OCR was 60% of maximum (KW chi = 73.9, p<0.0001) (Fig 4C). Astrocyte cultures also diverged in their response to EtBr, which reduced basal OCR much more than in NECos or neurons, and introduced a spare capacity into astrocyte mitochondrial respiration (Fig 4C).

### Astrocytes can respond to mitochondrial impairment by increasing glycolytic flux

**Fig 5:**
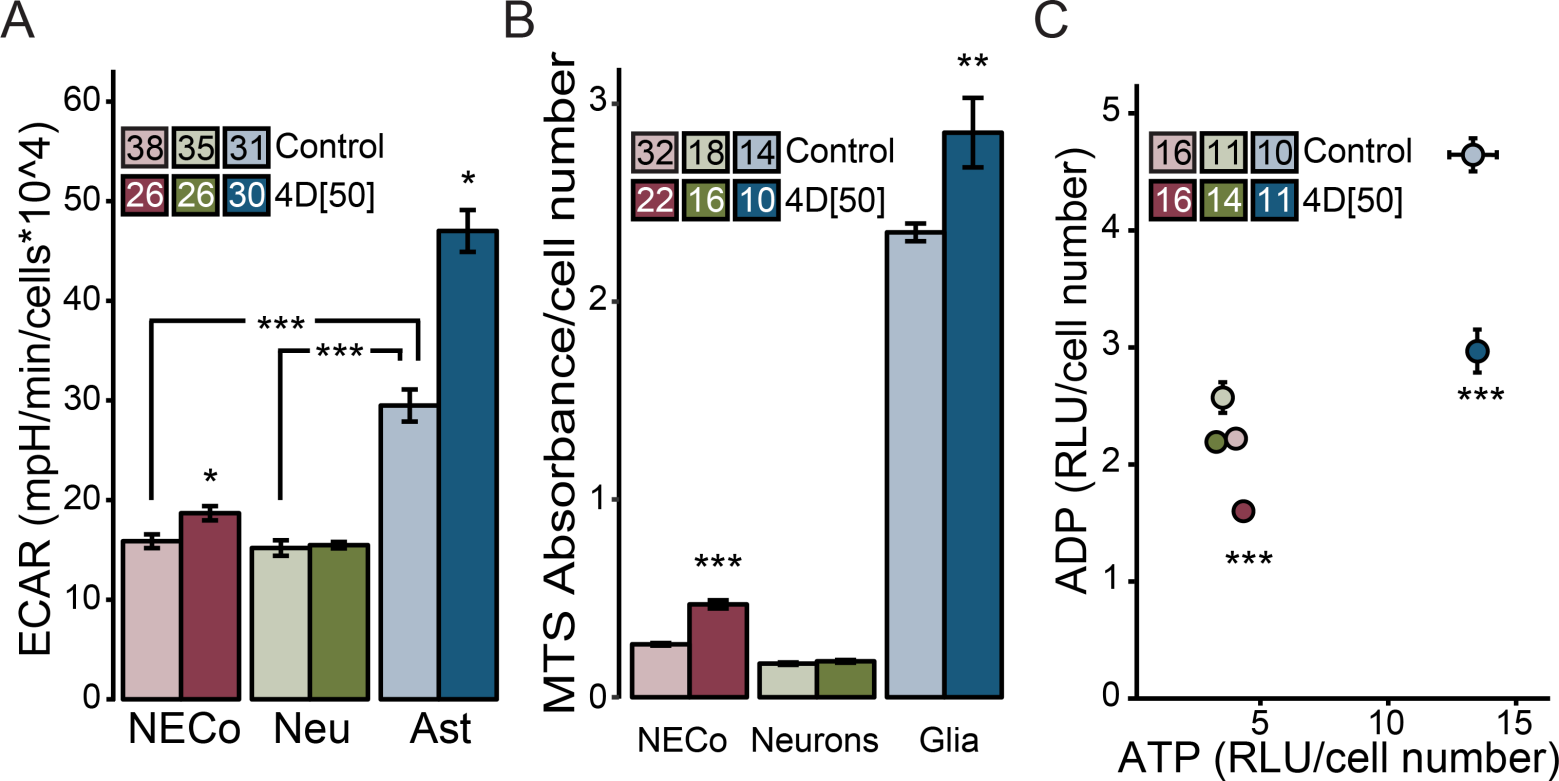
Impaired mitochondrial respiration increases astrocytic, but not neuronal, rate of glycolysis. (A) Basal extracellular acidification rate (ECAR) of NECos, neurons, and astrocytes, in the presence and absence of EtBr. (B) Relative quantity of electron donors (i.e. NAD(P)H, FADH_2_) across culture conditions, measured by MTS absorbance and normalized to cell number. (C) Relative ADP plotted against relative ATP. Arrows show the direction of change from control to EtBr treatment for each culture condition. RLU, relative luminescence units. Error bars reflect +/-SEM. N for each group is included in figure legends. (A) *** = p < 0.001; * = p < 0.05, following Dunn’s post-hoc test. (B), (C) *** = p < 0.001, ** = p < 0.01 compared to respective controls.

The reintroduction of a mitochondrial ‘spare capacity’ in astrocytes may be partially explained by the change observed in basal extracellular acidification rate (ECAR), recorded simultaneously with OCR. Following EtBr treatment, astrocyte basal ECAR increased by nearly 60% (Fig 5A), (KW chi = 114.31, p<0.0001), suggesting a shift from oxidative phosphorylation to glycolysis for ATP production. Similarly, NECo ECAR increased by 18%. In contrast, EtBr treatment of neurons did not change basal ECAR. ECAR of untreated astrocytes was also over 90% greater than the ECAR of untreated neurons, and 86% greater than the ECAR of untreated NECos, suggesting that the rate of astrocyte glycolytic glucose catabolism exceeds that of neurons.

As mitochondrial Complex I is a major source of NADH consumption via its oxidation to NAD+, we hypothesized that a reduction in mitochondrial respiratory chain activity could to lead to NADH accumulation. In NECo cultures, decreased mtDNA was accompanied by increased absorbance in an electron donor-dependent assay (Fig 5B). This was similarly observed in astrocyte but not neuronal cultures (Fig 5B). Likewise, EtBr treatment of NECos and astrocytes increased NADH without changing NAD+ levels in a specific NAD+/NADH assay (S3 Fig).

Decreased mitochondrial function also perturbed the ADP-ATP balance of NECos and astrocytes (Fig 5C). EtBr reduced ADP with little effect on ATP, and significantly changed the ATP/ADP ratio. In NECo cultures, EtBr raised the ratio from 1.8 to 2.7, and in astrocytes, the ratio increased from 2.8 to 4.8. In contrast, the ATP/ADP ratio in untreated neurons was 1.4, and 1.5 following EtBr treatment.

### Neurons, but not astrocytes, respond to mitochondrial impairment with a disruption in the phosphocreatine-creatine shuttle

While our data indicate that astrocytes can increase glycolytic activity in response to decreased mitochondrial respiration, neurons do not appear to have that capability. However, despite EtBr significantly reducing mitochondrial OCR, we did not observe significant alterations in the neuronal ATP/ADP ratio. We hypothesized that an alternate pathway might be utilized to maintain ATP levels specifically in neurons. Previous studies in other neurological disorders have linked mitochondrial dysfunction to perturbations in creatine kinase gene expression [71,72] and phosphocreatine (PCr) utilization [73,74], and our RNASeq data showed a downregulation of mtCK in the group of 20 most significantly regulated genes in neurons (S3 Table). Creatine kinase isoforms and the creatine transporter are differentially expressed in neurons and glia [11,75,76], which could allow for cell-type specific mechanisms to regulate the phosphocreatine-creatine (PCr-Cr) shuttle. We were therefore interested in quantifying Cr and PCr with ^1^H-NMR.

**Fig 6:**
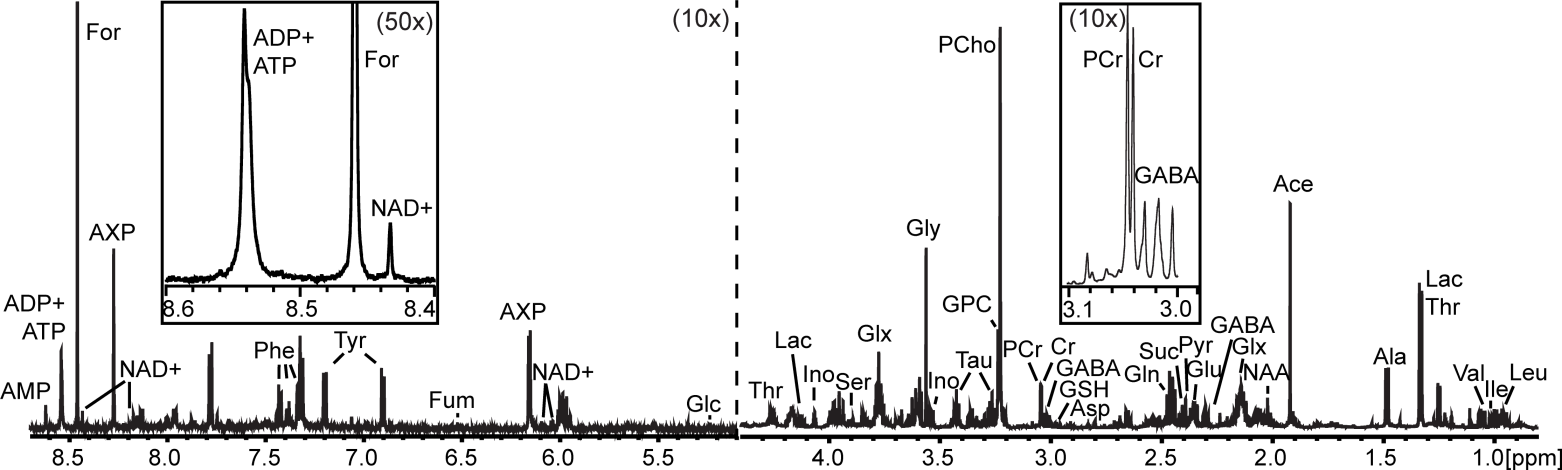
Representative NECo ^1^H NMR spectra. ^1^H NMR spectra at 600MHz from control NECo cultures from 0.8–4.5 ppm and from 5.0–8.8 ppm. 5.0–8.8 ppm section is magnified 10x relative to 0.8–4.5 ppm section. Leu–leucine; Ile–isoleucine; Val–valine; Lac–lactate; Thr–threonine; Ala–alanine; Ace–acetate; NAA–N-acetyl aspartate; Glx–glutamine/glutamate; GABA- gamma-amino butyrate; Glu–glutamate; Pyr–pyruvate; Suc–succinate; Gln–glutamine; Asp–aspartate; Cr–creatine; PCr–phosphocreatine; PCho–phosphocholine; GPC–glycerophosphocholine; Tau–taurine; Gly–glycine; Ser–serine; Ino–myoinositol; Glc–glucose; Fum–fumarate; Phe–phenylalanine; Tyr–tyrosine; AXP–combined adenine nucleotides; For–formate. Acetate and formate peaks are contaminants from sample preparation. Insets–Metabolites quantified for this study. Right, PCr and Cr peaks, 10x magnified. Left, ADP+ATP peak, 50x magnified.

**Fig 7:**
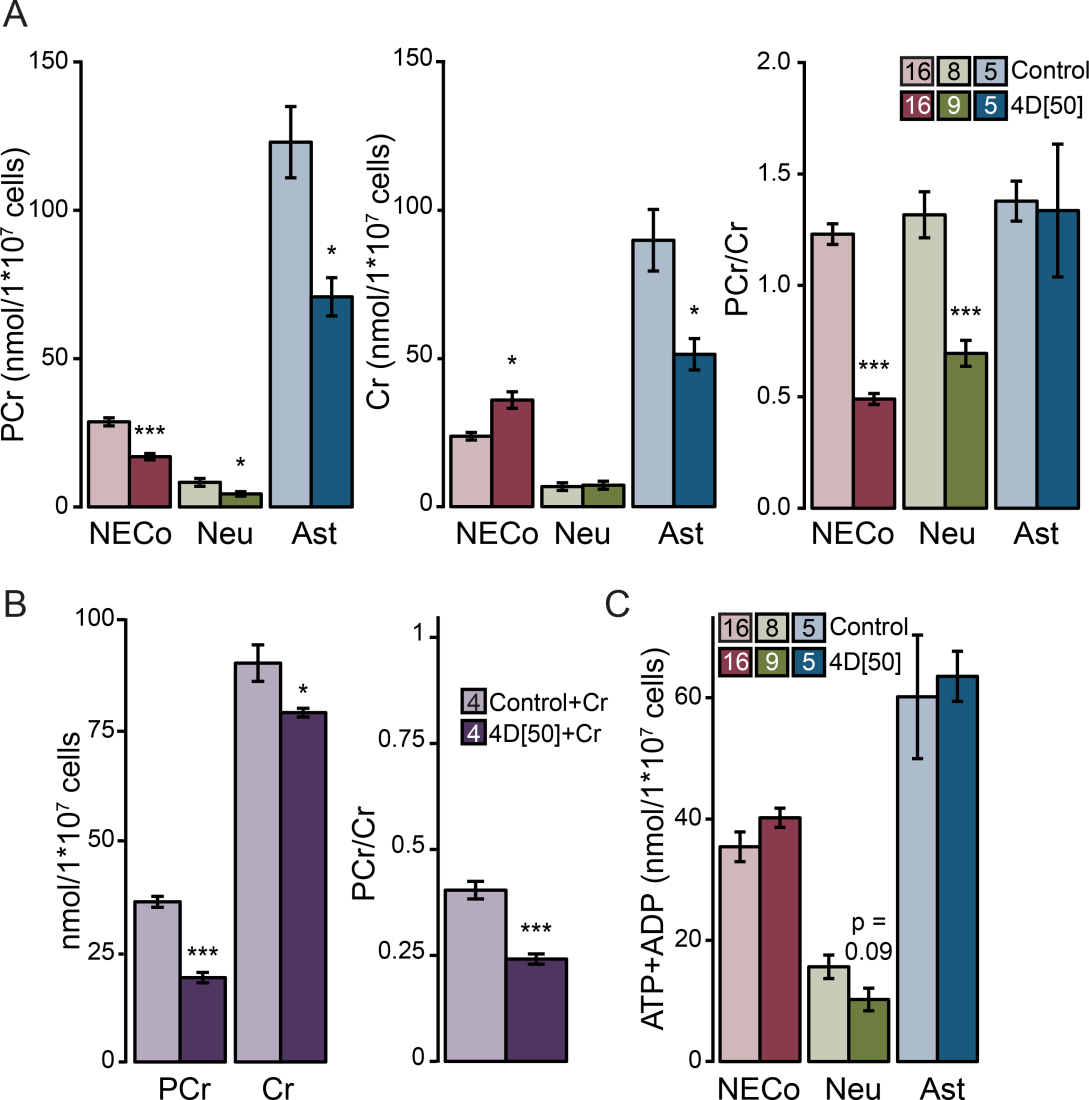
Reduced mitochondrial function decreases neuronal PCr/Cr ratio, and decreases glial tCr. (A) Phosphocreatine (PCr), creatine (Cr), and PCr/Cr ratio in NECos, neurons, and astrocytes, with and without EtBr. (B) Absolute PCr, Cr, and PCr/Cr ratio in neuronal cultures supplemented with 5 mM Cr 24h before harvest, with and without EtBr. (C) ATP+ADP in NECos, neurons, and astrocytes, with and without EtBr. N for each group is included in figure legends. Error bars reflect +/-SEM. *** = p< 0.001; ** = p < 0.01; * = p < 0.05, compared to respective controls.

Fig 6 shows a representative ^1^H-NMR spectrum from NECo cultures. We identified 30 different metabolites with a targeted metabolomic approach in NECo extracts, and quantified Cr, PCr, and the combined ADP+ATP peak in NECo, neuron, and astrocyte cultures. EtBr significantly decreased the amount of PCr, increased the amount of Cr, and decreased the PCr/Cr ratio in NECos (Fig 7A). Neurons similarly had a significant reduction in PCr and PCr/Cr ratio, though no change in Cr. In astrocytes, PCr and Cr were significantly reduced, with no change in the ratio. Because Cr in the brain is predominantly synthesized in astrocytes [11], the neuronal cultures had a very low basal concentration of Cr. To determine if low Cr might affect the ability of neurons to phosphorylate Cr, we supplemented neuronal cultures with 5 mM Cr 24h before harvest. While the total amount of Cr and PCr increased considerably, EtBr-treated cultures still had significantly decreased PCr, and a significant decrease in PCr/Cr ratio (Fig 7B). The amount of ATP+ADP was not different after EtBr treatment in any cell type (Fig 7C), which demonstrates that the reduction in ADP observed in NECos and astrocytes does not reflect a change in the total ATP+ADP pool.

**Fig 8:**
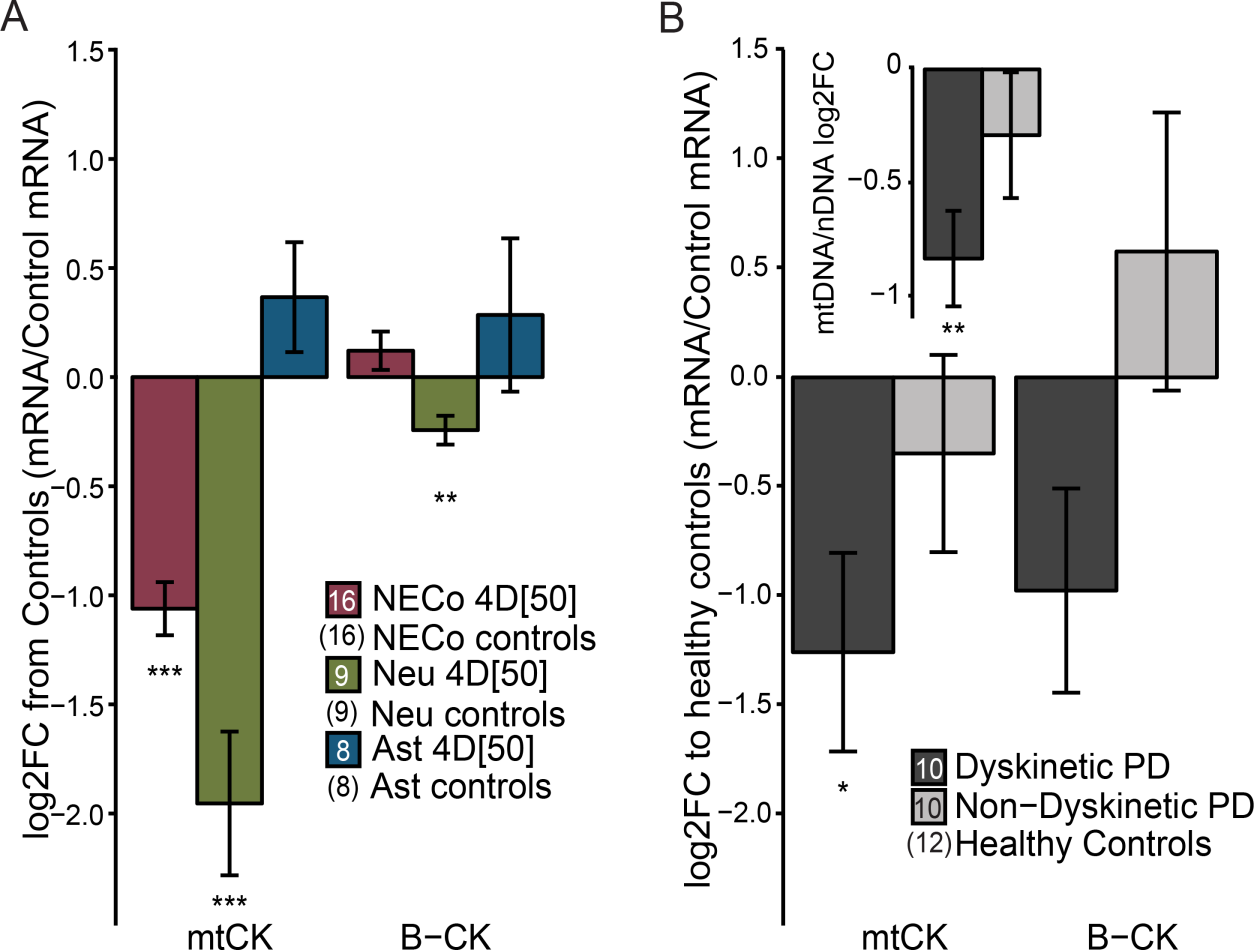
Decreased mtDNA quantity corresponds to decreased expression of mtCK. (A) Log2-fold change of mtCK and B-CK expression in rat NECos, neurons, and astrocytes, normalized to reference genes. (B) Log2-fold change of mtCK and B-CK expression in matched human dyskinetic and non-dyskinetic PD patients, normalized to reference genes and relative to control subjects. Inset: Relative mtDNA quantity from the same cohort as in (B), normalized to nDNA [43]. (A) *** = p < 0.001; ** = p < 0.01, relative to respective controls. (B, inset). ** = p < 0.01, * = p < 0.05, following Tukey’s HSD post-hoc test.

We further used qPCR to examine the expression of genes involved in the regulation of the PCr/Cr pool–the mitochondrial mtCK, and the brain isoform of the cytosolic creatine kinase, B-CK. EtBr treatment decreased expression of mtCK (Fig 8A) in NECos and neuron cultures, but not in astrocytes. B-CK was slightly reduced in neuronal cultures. Despite reports of the segregation of mtCK into neurons, and B-CK into astrocytes [11,75], we detected expression of both genes in both culture types. Data from RNASeq experiments supported the qPCR data (S5 Fig).

Since we have previously reported mtDNA depletion in the putamen of PD patients with LID [43], we sought to determine if this was also associated with downregulation of mtCK gene expression, in keeping with our observations in EtBr-treated primary neuronal cultures. Indeed, we found significantly decreased mtCK mRNA levels in the putamen of PD patients with LID compared to controls (F = 3.722, p = 0.03) (Fig 8B) (see S1 Table for demographic data). Neither mtCK nor B-CK was significantly different from control in non-dyskinetic PD patients. Further, we confirmed that these LID patients showed a reduction in mtDNA levels (F = 5.546, p<0.01) (Fig 8B inset) (partial sample overlap with previously published data [43]).

## Discussion

Decreased mtDNA has been identified as a factor in multiple neurological disorders. In our lab, we previously observed that PD patients who developed LID had less mtDNA than their non-dyskinetic counterparts [43]. Here, we examined how mtDNA reduction affects different elements of energy homeostasis in striatal neurons and astrocytes. First, we validated the use of EtBr to model mtDNA reduction in primary neuronal and/or astrocyte cultures. Second, we demonstrated that astrocytes can increase glycolytic activity in response to mitochondrial dysfunction, supporting previous studies [77,78]. Finally, we observed a relationship between mtDNA, mtCK expression, and PCr levels in neurons, and showed that the decrease in mtCK expression observed after mtDNA loss is also observed in PD patients with LID.

### The utility of EtBr in modeling mtDNA and mtRNA reduction

EtBr accumulates in mitochondria, where it intercalates into mtDNA and interferes with replication [38] and transcription [79]. EtBr is frequently employed to generate rho0 cell lines, which lack mtDNA, and as a powerful and rapid means to manipulate mtDNA and mtRNA quantities [28,57,80,81]. Although astrocyte mtDNA was less sensitive to EtBr than neuronal mtDNA, doses of EtBr that had no significant effect on mtDNA significantly reduced mtRNA in both cell types. This was most likely due to EtBr intercalating with and occluding mtDNA, which affects both mtDNA replication and transcription [38]. Because of the polycistronic nature of mtRNA transcription, premature transcription cessation caused by EtBr intercalation into mtDNA should more dramatically affect promoter-distal mtRNAs [82]. This was supported by our observation that mtRNAs more distal from the mtDNA heavy strand promoter were more reduced by EtBr (Fig 3).

### Reduced mtDNA and mtRNA decreases mitochondrial respiration and increases glycolytic flux in astrocytes

Basal, maximum, and leak respiration were reduced after EtBr treatment in all culture conditions. Despite the lower sensitivity of astrocyte mtDNA to EtBr, the reduction in mtRNA was sufficient to reduce mitochondrial OCR. Despite the significant reduction in OCR, we did not observe a reduction in viability following EtBr treatment.

Astrocyte basal respiration was equal to its maximum, in contrast to neurons, which had a significant spare capacity. This difference has also been observed previously and might be characteristic for resting neurons [83]. Following EtBr treatment, astrocyte basal respiration was significantly lower than its maximum, a reinstatement of spare capacity that was unexpected but could be a consequence of a switch to alternative metabolic pathways. ATP production in astrocytes may have been shunted away from mitochondrial respiration toward glycolytic flux, demonstrated by an increase in astrocyte ECAR, an increase in NADH, and an increase in glucose consumption. This is in line with previous observations about the capacity of astrocytes to increase the rate of glycolysis, which is mediated by PFKFB3 [7,84]. Increased expression of PFKFB3 increases production of fructose-2,6-bisphosphate, which stimulates phosphofructokinase, a rate-limiting enzyme in glycolysis. In neurons PFKFB3 is constitutively degraded, which precludes upregulation of glycolysis [7].

### Reduced mtDNA diminishes PCr levels in neurons

Mitochondrial function is vital for maintaining intracellular phosphate balance, which is particularly important for neurons [85]. Our results showed that EtBr-reduced mtDNA affects mitochondrial oxygen consumption, which should limit ATP production. Neurons compensate by a downregulation of mtCK and PCr synthesis to sustain basal ATP levels.

Mitochondrially-generated ATP is exported from the mitochondrial matrix by the adenine nucleotide transporter (ANT) and directly targeted by mtCK. mtCK resides in the intermembrane space of mitochondria, where it transfers a phosphate from ATP to Cr to form PCr and ADP. Under high glycolytic ATP production, B-CK may function as a kinase as well as a phosphatase, interconverting Cr and PCr [10]. In contrast, the high concentration of ATP near mtCK renders it almost exclusively a kinase. A downregulation of mtCK should consequently decrease the levels of PCr, whereas changes in B-CK expression could have more complex effects on the PCr/Cr ratio. Accordingly, we observed both a reduction in mtCK expression and PCr. Downregulation of mtCK has also been observed in a number of cancers [86,87], where increased energetic demand of tumor cells may require the immediate use of all ATP and disfavor the synthesis of PCr.

The Cr/PCr system functions as a temporal and spatial energy buffer of vital importance for synaptic activity, membrane polarization, and overall cellular maintenance. Cytosolic creatine kinase is associated with synapses and with membrane ATPases such as ion pumps in the plasma membrane, where it can quickly convert PCr to Cr and ATP to maintain network activity [88]. A shortage of PCr at sites of high ATP demand may contribute to the deregulation of striatal and other neurons, as it becomes difficult from a bioenergetics standpoint to regulate ion channels, membrane potentials, and vesicle release [89]. To our knowledge, this study is the first to demonstrate a connection between reduced mitochondrial function and downregulation of mtCK in neurons. As both the relationship between mtDNA quantity and energy metabolism and the relationship between mitochondrial function and mtCK expression are controversial, we believe that additional studies utilizing other methods of mtDNA reduction would be beneficial. We hypothesize that a reduction in the efficacy of the Cr-PCr shuttle could be an important metabolic perturbation arising from reduced mtDNA.

### Extrapolating the relationship between mtDNA quantity and mtCK to models of PD and LID

In this study we used EtBr to reduce mtDNA to understand the physiological consequences of low mtDNA levels observed in PD patients with LID. Future studies should address the interaction between mtDNA, PCr, L-DOPA treatment, and LID. Transcriptomic [90] and proteomic [91] studies have shown that mtCK is downregulated in LID animals, while Cr supplementation improved LID-associated motor disturbances [92]. These studies do not address if L-DOPA caused the downregulation of mtCK, or if lower Cr levels lead to increased sensitivity to develop LID. For example, although L-DOPA does not seem to decrease mtDNA in the striatum [43], it might lead to LID in PD patients who have a priori lower mtDNA levels.

Multiple tools could be useful to further explore the cause and effect of mtDNA, PCr, and L-DOPA in the pathogenesis of LID. Among them are rotenone and MPTP which inhibit mitochondrial Complex I and oxidatively damage mtDNA [93,94]; a knockdown model of *Tfam* which reduces mtDNA quantity without damaging the nuclear genome [27]; and the development of an animal model of reduced striatal mtDNA, analogous to the MitoPark mouse [95]. It will be important to assess the effect of L-DOPA on these models of reduced mtDNA, and determine how L-DOPA may affect Cr homeostasis or, conversely, how Cr homeostasis might shape vulnerability to LID.

## Conclusions

Maintenance of mitochondrial function is important for the brain and for neurons, due to their high demand for ATP. Post-mortem studies have associated reduced mtDNA copy number with multiple neurological disorders, but post-mortem studies preclude most functional analyses. We demonstrate that reducing mtDNA by EtBr in rat primary cultures decreases mtRNA and respiration, increases glycolysis in glia, and perturbs the neuronal phosphate balance by decreasing mtCK expression and Cr phosphorylation in neurons. We additionally show that mtCK is significantly downregulated in patients with LID. These observations enhance our understanding of how decreased mtDNA in the putamen may be related to the development of LID, and how reduced mtDNA could more generally contribute to energetic instability in the brain.

## Supporting information

S1 Fig EtBr treatment increases NECo and astrocyte glucose consumption. Representative traces from single dissections of NECo (left), neuron (middle), and astrocyte (right) media glucose concentrations from plating until harvest. Beginning DIV2, media glucose was monitored daily and restored to 2.5 mM. Glucose concentrations decreased more rapidly in astrocyte and NECo cultures under EtBr treatment. N for each group is included in figure legends. Error bars reflect +/-SEM.
(TIF)

S2 Fig Heat maps of all genes with 5% coefficient of variation cluster EtBr-treated samples from vehicle-treated samples. (A) NECo cultures, (B) neuron cultures, (C) astrocytes. All cultures were treated with EtBr (50ng/ml) for 4 days. Each sample was pooled from samples from two independently dissected culture experiments and subjected to RNASeq.
(TIF)

S3 Fig EtBr decreases NAD+ and increases NADH and NADH/NAD+ ratio in NECos and astrocytes. Left, Absolute NAD+ and NADH concentration in NECo and glia cultures (nM); Right, NADH/NAD+ ratio for NECo and glia cultures. Both experiments are from a single dissection. N for each group is included in figure legends. * = p < 0.05; ** = p < 0.01; *** = p < 0.001, relative to controls. Error bars reflect +/- SEM.
(TIF)

S4 Fig A high dose of EtBr significantly reduces mtDNA, mtRNA, and increases mtCK in glia. Log2-fold change in mtDNA and mtRNA quantity in glial cultures in response to 500 ng/mL EtBr for 4 days (4D[500]), measured with qPCR. Single dissection, n = 6. Expression was normalized to nDNA (for mtDNA) or reference genes (for mtRNA). ** = p < 0.01; *** = p < 0.001, relative to controls. Error bars reflect delta-method propagated +/-SEM. Baseline (0 on y-axis) reflects control levels.
(TIF)

S5 Fig Expression levels of mtCK and CK-B in NECos, neurons and glia in RNASeq experiment. RNASeq data, analyzed with DESeq2, mirror qPCR data in Fig 7A. N = 3 samples per group, each sample pooled from two different dissections. *** = adjusted p < 0.001, as calculated with DESeq2.
(TIF)

S1 Table Demographic data of human putamen samples. All samples are male.
(PDF)

S2 Table Primer sequences and efficiencies. (PDF)

S3 Table RNASeq analysis of genes most significantly regulated by EtBr treatment. The 20 genes with the strongest regulation, based on ranking in DESeq2 (highlighted in brown), are listed in striatal NECos, purified neuronal cultures, and astrocytes. Each row is a comparison of three control samples to three samples treated with 50ng/ml EtBr for 4 days, each sample pooled from two independently dissected culture experiments. In neuronal cultures, most highly-regulated genes were encoded in mtDNA, and downregulated. A similar, albeit lesser, trend was observed in NECos. Any mtDNA-derived gene listed in the sequencing results but not in the group of 20 is added below each table for completion. Because the 18 strongest regulated genes in neuronal culture had a p-value below 1*10^−307^ and thus could not be ranked individually, an average ranking number ("8" in DESeq,"9" in edgeR) was assigned to each one. This was therefore the lowest possible number. Although the majority of these strongest regulated genes were downregulated, the overall percentage of downregulated and upregulated genes throughout each dataset was equivalent (S4 Table).
Of note, mitochondrial creatine kinase and amino acid transporters were in the group of highest regulated genes in neurons.
(PDF)

S4 Table Distribution of the most significantly up- and downregulated genes analyzed with MultiRankSeq. The 2000, 1000, and 500 genes most significantly regulated by EtBr treatment in all three culture conditions were assessed for directionality of regulation. Across conditions, significantly regulated genes were approximately evenly distributed between up- and downregulated groups.
(PDF)

S5 Table NIH DAVID analysis of RNASeq data. NIH DAVID was used for pathway analysis of RNASeq data, with focus on the UniProt and KEGG databases, which have in-depth characterization of gene groups [69]. The 500 strongest downregulated and 500 strongest upregulated genes after EtBr treatment (50 ng/ml, 4 days), ranked with DESeq2, which together made up less than 5% of all genes, were used in separate analyses.
Category = Original database
Term = Enriched terms associated with input gene list
Count = Genes involved in term
% = involved genes/total genes
PValue = modified Fisher’s exact p-value
Bonferroni = Bonferroni-corrected p-value
Benjamini = Benjamini-Hochberg corrected p-value
FDR = False discovery rate
(PDF)
