# Supplementary figures and images for: Mitochondrial DNA depletion by ethidium bromide decreases neuronal mitochondrial creatine kinase: Implications for striatal energy metabolism
Source: PLoS One. 2017 Dec 29;12(12):e0190456. doi: 10.1371/journal.pone.0190456 (PMC5747477; doi:10.1371/journal.pone.0190456)

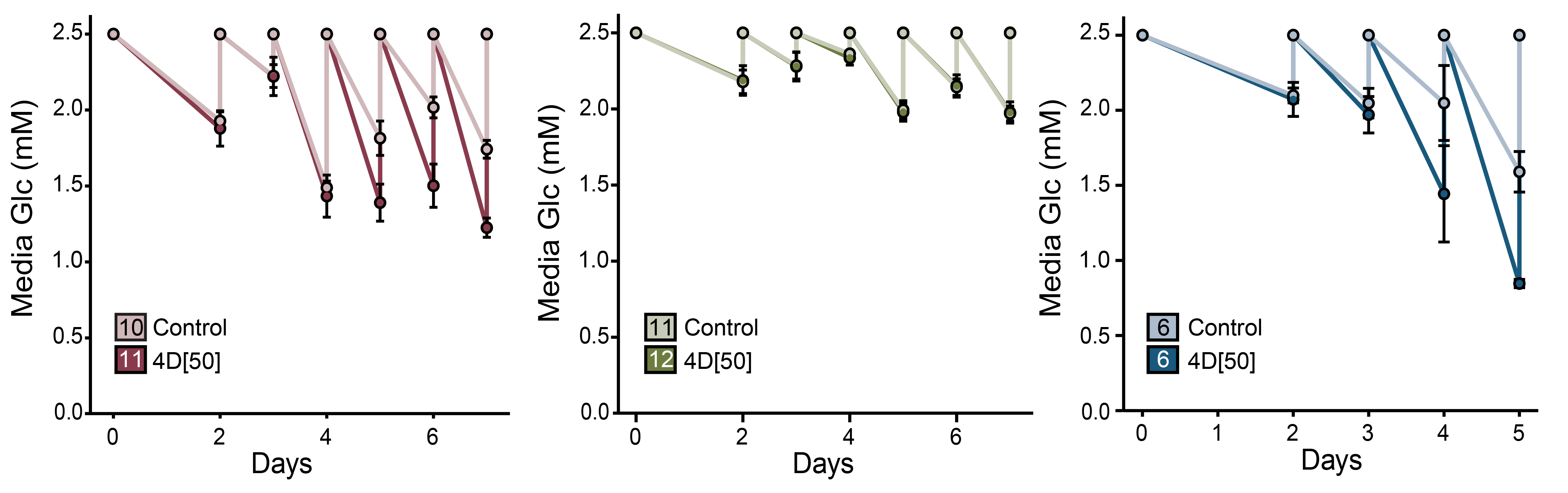

Supplement: S1 Fig — Representative traces from single dissections of NECo (left), neuron (middle), and astrocyte (right) media glucose concentrations from plating until harvest. Beginning DIV2, media glucose was monitored daily and restored to 2.5 mM. Glucose concentrations decreased more rapidly in astrocyte and NECo cultures under EtBr treatment. N for each group is included in figure legends. Error bars reflect +/-SEM. (TIF) [file pone.0190456.s001.tif]

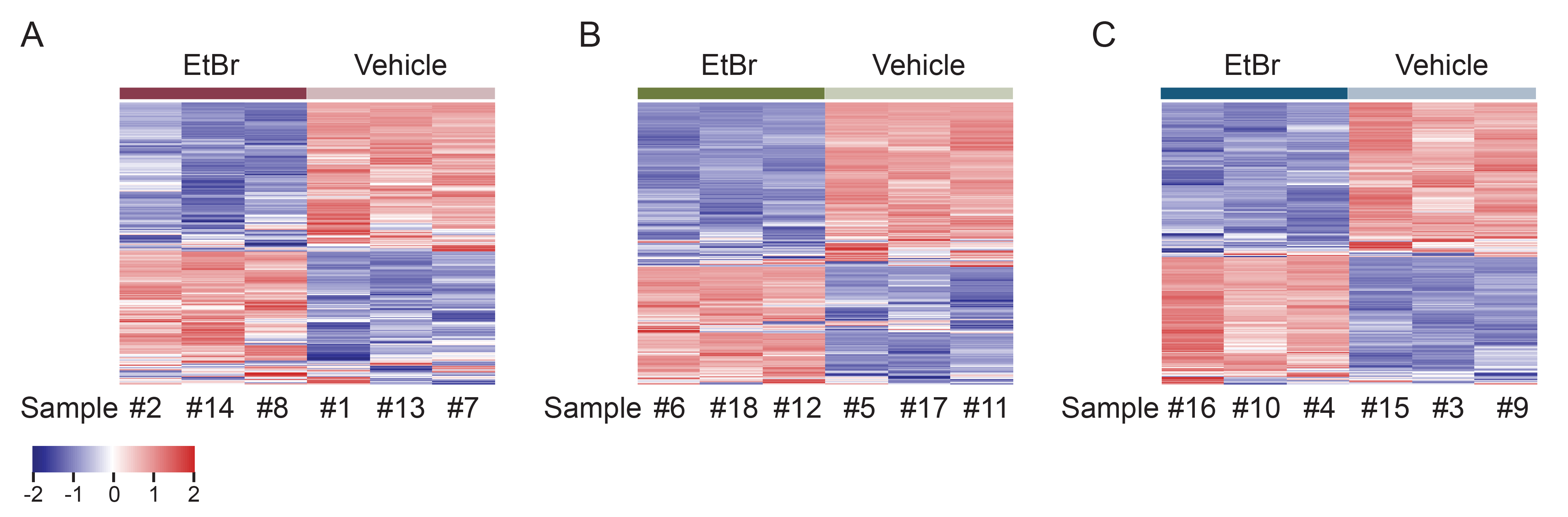

Supplement: S2 Fig — (A) NECo cultures, (B) neuron cultures, (C) astrocytes. All cultures were treated with EtBr (50ng/ml) for 4 days. Each sample was pooled from samples from two independently dissected culture experiments and subjected to RNASeq. (TIF) [file pone.0190456.s002.tif]

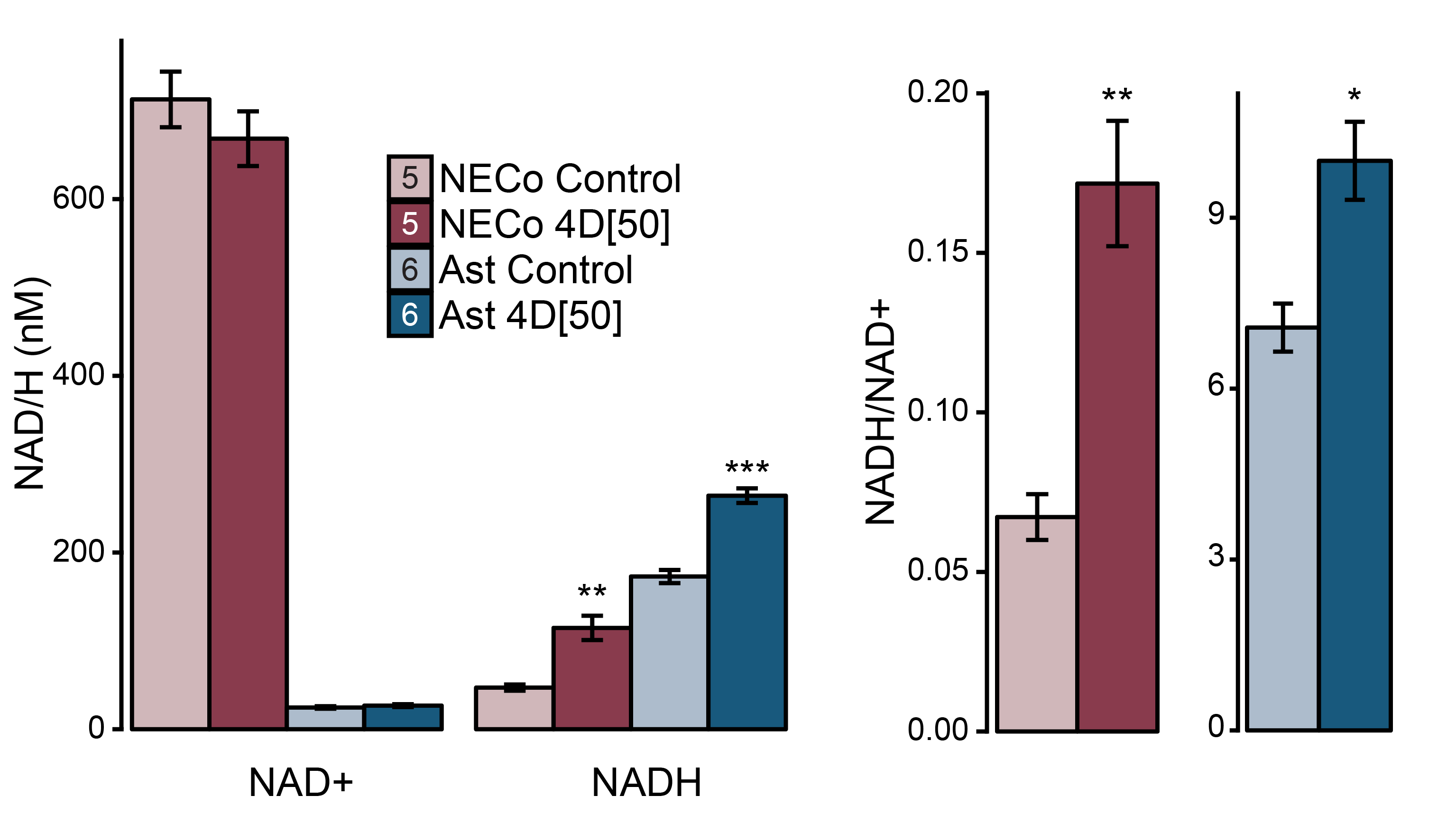

Supplement: S3 Fig — Left, Absolute NAD+ and NADH concentration in NECo and glia cultures (nM); Right, NADH/NAD+ ratio for NECo and glia cultures. Both experiments are from a single dissection. N for each group is included in figure legends. * = p < 0.05; ** = p < 0.01; *** = p < 0.001, relative to controls. Error bars reflect +/- SEM. (TIF) [file pone.0190456.s003.tif]

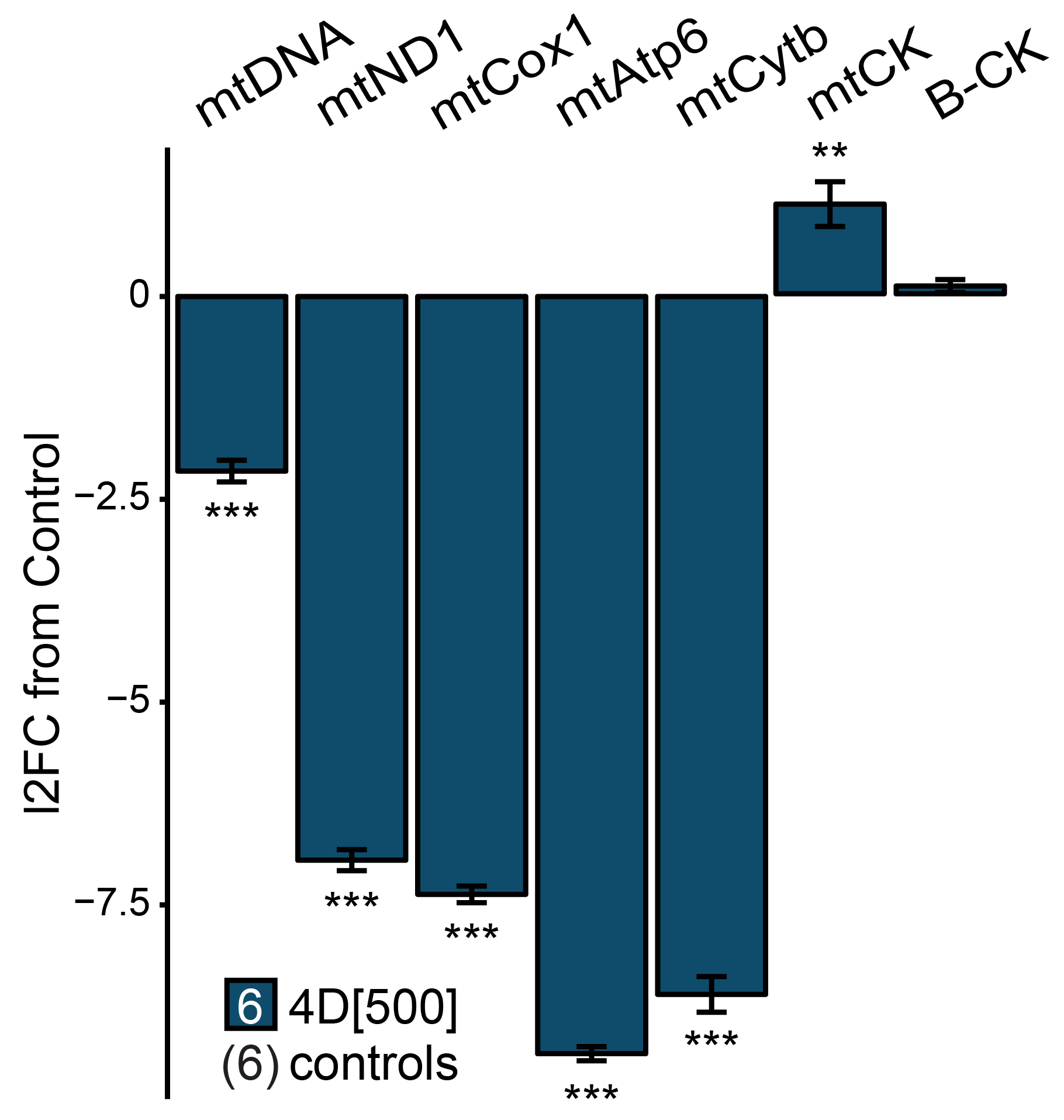

Supplement: S4 Fig — Log2-fold change in mtDNA and mtRNA quantity in glial cultures in response to 500 ng/mL EtBr for 4 days (4D[500]), measured with qPCR. Single dissection, n = 6. Expression was normalized to nDNA (for mtDNA) or reference genes (for mtRNA). ** = p < 0.01; *** = p < 0.001, relative to controls. Error bars reflect delta-method propagated +/-SEM. Baseline (0 on y-axis) reflects control levels. (TIF) [file pone.0190456.s004.tif]

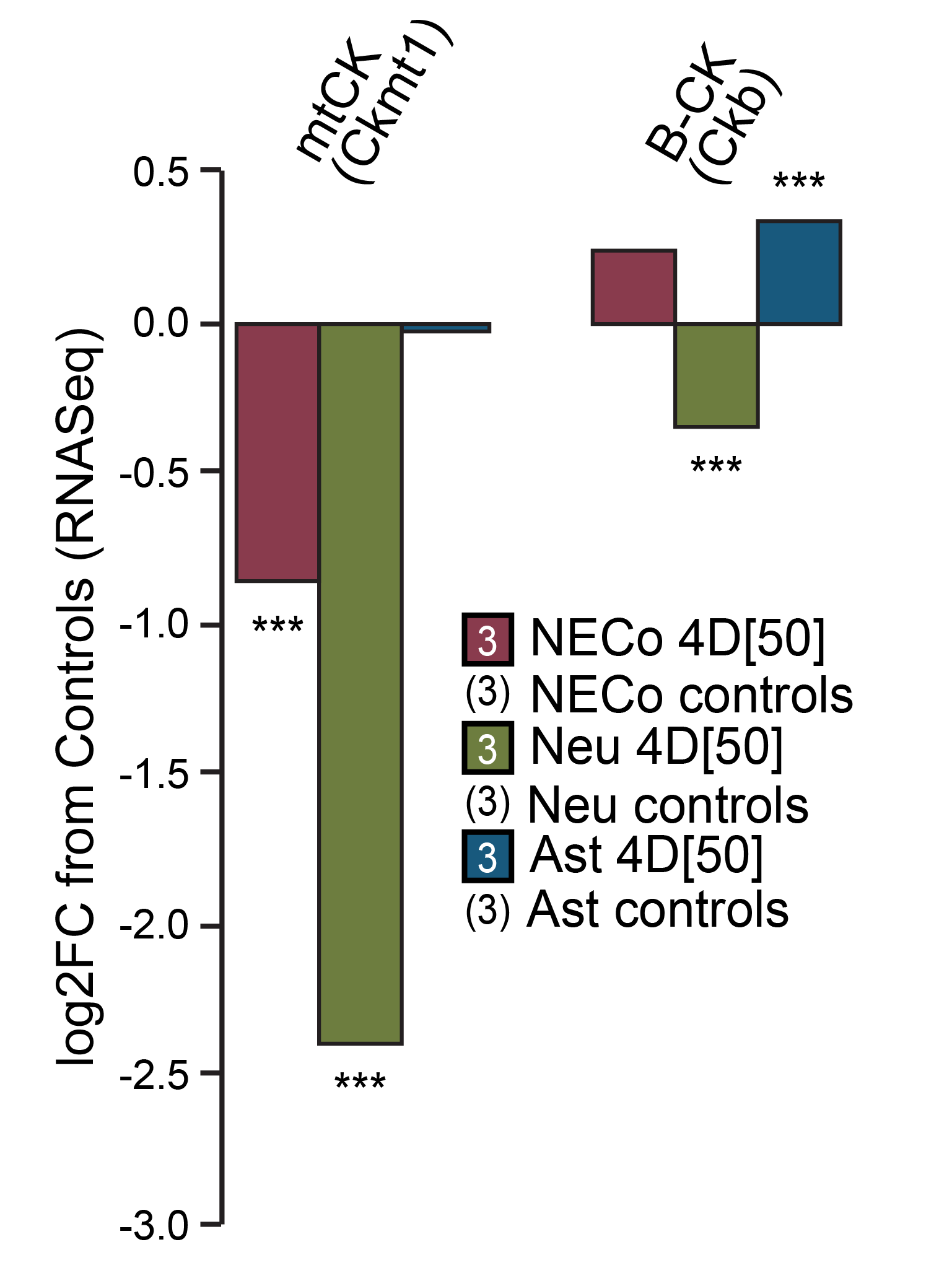

Supplement: S5 Fig — RNASeq data, analyzed with DESeq2, mirror qPCR data in Fig 7A. N = 3 samples per group, each sample pooled from two different dissections. *** = adjusted p < 0.001, as calculated with DESeq2. (TIF) [file pone.0190456.s005.tif]
