# Supplementary material for: Mitochondrial DNA depletion by ethidium bromide decreases neuronal mitochondrial creatine kinase: Implications for striatal energy metabolism
Source: PLoS One. 2017 Dec 29;12(12):e0190456. doi: 10.1371/journal.pone.0190456 (PMC5747477; doi:10.1371/journal.pone.0190456)
Supplement: S1 Table — All samples are male. (PDF) [file pone.0190456.s006.pdf]

S1 Table. Demographic data of human putamen samples

Diagnosis	n=	Age	PMI	Years diagnosed	Fresh brain weight (g)	Treatment duration (y)	Average levodopa per year (g)	SN-pigmentation (pallor)[1]	SN-cell loss[2]
control	12	74.7±3.6	16.6±4.4		1304.6±138.6				
dyskinesia	10	76.5±7.5	12.2±6.9	11.8±4.6	1300.6±59.8	7.9±5.3	174.7±74.8	3.3±0.7	2.7±0.3
nondyskinesia	10	75.5±2.3	16.1±5.9	8.0±3.1	1370.8±178.4	5.1±2.8	169.1±72	2.8±0.7	2.5±0.8

[1] Scale from 0-4;   All samples male
[2] Scale from 0-3
