# Supplementary material for: Mitochondrial DNA depletion by ethidium bromide decreases neuronal mitochondrial creatine kinase: Implications for striatal energy metabolism
Source: PLoS One. 2017 Dec 29;12(12):e0190456. doi: 10.1371/journal.pone.0190456 (PMC5747477; doi:10.1371/journal.pone.0190456)
Supplement: S2 Table — (PDF) [file pone.0190456.s007.pdf]

S2 Table. Primer sequences and efficiencies

Rat Primers:	Sequence	DNA/RNA	Efficiency
mtCox1 F<br>mtCox1 R	AGGAGCAGTATTCGCCATCA<br>CGACGAGGTATCCCTGCTAA	DNA + RNA	DNA - 105%<br>RNA - 100%
mtCytb F<br>mtCytb R	CGCCCCATCTAACATCTCAT<br>GTCCCACATGGAGGAATAGG	DNA + RNA	DNA - 115%<br>RNA - 101%
mtND1 F<br>mtND1 R	CCTCACCCCCTTATCAACCT<br>GGAGCCGCTTATTAGGAGGA	DNA + RNA	DNA - 101%<br>RNA - 100%
mtATP6 F<br>mtATP6 R	CACACCAAAAGGACGAACCTGA<br>CTGCTCATAGGGGGATGGCT	RNA	101%
Tuba1a F<br>Tuba1a R	ACCAAGCGTACCATCCAGTT<br>CCACGTACCAGTGCACAAAG	DNA	97%
Crh F<br>Crh R	GCTAACTTTTTCCGCGTGTT<br>GGTGGAAGGTGAGATCCAGA	DNA	97%
Pdyn F<br>Pdyn R	GCAGGAAGCCCCCATAGC<br>CGCAAATACCCCAAGAGGAG	DNA	92%
mtCK F<br>mtCK R	GAATGAGGAGGACCACACACG<br>CAGTGCCCAGGTTAGATGGAC	RNA	83%
B-CK F<br>B-CK R	AACAGCCACAACACGCAGAA<br>TCGTAACTCTCCTCGTCGCC	RNA	97%
Canx F<br>Canx R	TGACCCCTCCGGTAAACCCT<br>CCTCCCATTCTCCGTCCATATCC	RNA	93%
18S F<br>18S R	TGGCTCAGCGTGTGCCTACC<br>TAGTAGCGACGGGCGGTGTG	RNA	104%

Human Primers:	Sequence	DNA/RNA	Efficiency
mtCK F<br>mtCK R	ACATCAAACTGCCCCTGCTA<br>ATCAAAGACACCGCCTGTAG	RNA	107%
B-CK F<br>B-CK R	CGGTATCTGGCACAATGACA<br>ATGGGCAGGTGAGGATGTAG	RNA	99%
Canx F<br>Canx R	ACTTGTGTTGATGTCTCGGGC<br>CGTTTTGGGGTTTTTGTGTCGG	RNA	85%
Itm2b F<br>Itm2b R	CATTGTTATGCCACCCAGAA<br>GCAGTTTGTAAGTTTCCTTGTCA	RNA	88%
