# Supplementary material for: Mitochondrial DNA depletion by ethidium bromide decreases neuronal mitochondrial creatine kinase: Implications for striatal energy metabolism
Source: PLoS One. 2017 Dec 29;12(12):e0190456. doi: 10.1371/journal.pone.0190456 (PMC5747477; doi:10.1371/journal.pone.0190456)
Supplement: S3 Table — The 20 genes with the strongest regulation, based on ranking in DESeq2 (highlighted in brown), are listed in striatal NECos, purified neuronal cultures, and astrocytes. Each row is a comparison of three control samples to three samples treated with 50ng/ml EtBr for 4 days, each sample pooled from two independently dissected culture experiments. In neuronal cultures, most highly-regulated genes were encoded in mtDNA, and downregulated. A similar, albeit lesser, trend was observed in NECos. Any mtDNA-derived gene listed in the sequencing results but not in the group of 20 is added below each table for completion. Because the 18 strongest regulated genes in neuronal culture had a p-value below 1*10−307 and thus could not be ranked individually, an average ranking number ("8" in DESeq,"9" in edgeR) was assigned to each one. This was therefore the lowest possible number. Although the majority of these strongest regulated genes were downregulated, the overall percentage of downregulated and upregulated genes throughout each dataset was equivalent (S4 Table). Of note, mitochondrial creatine kinase and amino acid transporters were in the group of highest regulated genes in neurons. (PDF) [file pone.0190456.s008.pdf]

S3 Table. RNASeq analysis of genes most significantly regulated by EtBr treatment.

Neuron-enriched Co-cultures

Ensemble Gene ID	Gene ID	log2Fold change (DESeq2)	pAdj (DESeq2)	rank (DESeq)	pValue (DESeq2)	log2Fold change (edgeR)	pValue (edgeR)	pAdj (edgeR)	rank (edgeR)	1-Likelihood (baySeq)	AdjLikelihood (baySeq)	log2Fold change (raw)	rank (baySeq)	Sum of ranks
ENSRNOG00000019598	Vegfa	1.20	2.09E-126	1	1.55E-130	1.28	1.64E-129	1.14E-125	3	0.00E+00	0.00E+00	1.37	17.5	21.5
ENSRNOG00000011921	Dusp4	-1.16	3.93E-79	2	5.80E-83	-1.21	1.43E-82	7.44E-79	4	0.00E+00	0.00E+00	-1.12	17.5	23.5
ENSRNOG00000007546	Asns	0.77	3.21E-67	3	7.11E-71	0.82	1.07E-67	2.80E-64	8	0.00E+00	0.00E+00	0.91	17.5	28.5
ENSRNOG00000018824	Slc7a5	0.72	1.63E-62	4	4.83E-66	0.76	1.25E-33	6.51E-31	40	0.00E+00	0.00E+00	0.85	17.5	61.5
ENSRNOG00000009740	Slco1c1	-0.89	1.10E-58	5	4.06E-62	-0.91	3.03E-66	6.31E-63	10	2.34E-11	1.28E-12	-0.82	58	73
ENSRNOG00000016552	Hmgcs1	0.60	4.15E-56	6	1.84E-59	0.64	1.19E-26	4.19E-24	59	0.00E+00	0.00E+00	0.73	17.5	82.5
ENSRNOG00000004290	Grb10	1.06	1.15E-53	7	5.96E-57	1.17	4.41E-67	1.02E-63	9	2.85E-08	1.47E-09	1.27	94	110
ENSRNOG00000003256	Ccng1	0.64	2.30E-49	8	1.36E-52	0.68	3.20E-44	3.04E-41	22	7.11E-15	2.09E-16	0.77	35.5	65.5
ENSRNOG00000029971	**Mt-nd5**	-1.90	3.37E-48	9	2.24E-51	-3.98	4.99E-142	5.20E-138	2	0.00E+00	0.00E+00	-3.89	17.5	28.5
ENSRNOG00000058645	Tnc	-0.69	5.47E-48	10	4.04E-51	-0.69	8.70E-23	2.11E-20	86	0.00E+00	0.00E+00	-0.60	17.5	113.5
ENSRNOG00000000521	Cdkn1a	0.86	4.14E-47	11	3.36E-50	0.93	4.10E-61	7.77E-58	11	2.06E-09	1.15E-10	1.03	81	103
ENSRNOG00000002393	Eprs	0.53	4.60E-47	12	4.07E-50	0.57	1.59E-26	5.44E-24	61	9.91E-06	9.06E-07	0.66	160	233
ENSRNOG00000028274	Myrf	-1.03	2.22E-45	13	2.13E-48	-1.10	3.79E-53	6.58E-50	12	3.06E-07	2.60E-08	-1.01	117	142
ENSRNOG00000037718	Cfap52	-1.47	2.84E-44	14	2.94E-47	-1.82	1.96E-52	3.14E-49	13	7.11E-15	4.06E-16	-1.74	35.5	62.5
ENSRNOG00000022162	Pbx3	-0.57	7.94E-40	15	8.79E-43	-0.56	4.50E-36	2.84E-33	33	2.57E-10	2.10E-11	-0.47	72	120
ENSRNOG00000029042	**Mt-nd6**	-1.70	8.50E-39	16	1.00E-41	-4.28	2.29E-162	4.77E-158	1	0.00E+00	0.00E+00	-4.19	17.5	34.5
ENSRNOG00000011305	Sox10	-0.98	6.71E-38	17	8.41E-41	-1.05	2.17E-40	1.67E-37	27	3.39E-07	2.87E-08	-0.97	118	162
ENSRNOG00000021318	Epas1	0.53	1.68E-36	18	2.24E-39	0.57	6.22E-29	2.65E-26	49	6.41E-05	5.86E-06	0.67	190	257
ENSRNOG00000029145	**Mt_tRNA**	-1.50	1.61E-34	19	2.26E-37	-2.20	1.18E-44	1.17E-41	21	1.21E-07	1.09E-08	-2.11	107	147
ENSRNOG00000014387	Chac1	0.69	1.07E-32	20	1.58E-35	0.75	1.01E-45	1.12E-42	18	1.92E-06	1.23E-07	0.85	135	173
**Other mitochondrial RNA**														
ENSRNOG00000043866	Mt_rRNA	-0.57	5.80E-29	27	1.16E-31	-0.57	4.11E-20	7.65E-18	112	9.94E-01	8.57E-01	-0.48	14588	14727
ENSRNOG00000030478	Mt_rRNA	0.93	1.27E-21	54	5.04E-24	1.13	9.37E-44	7.92E-41	24	4.30E-02	7.42E-03	1.22	612	690
ENSRNOG00000029707	Mt-nd4	-1.08	4.02E-18	84	2.49E-20	-4.48	1.35E-80	5.65E-77	5	4.41E-09	2.42E-10	-4.40	85	174
ENSRNOG00000030644	Mt-nd1	-1.04	1.82E-15	113	1.52E-17	-3.02	2.88E-52	4.28E-49	14	1.00E+00	8.99E-01	-2.93	20845.5	20972.5
ENSRNOG00000031766	Mt-cyb	-0.90	4.63E-14	130	4.44E-16	-4.95	7.00E-73	2.43E-69	6	1.00E+00	8.99E-01	-4.87	20832	20968
ENSRNOG00000031053	Mt-nd4l	-0.89	1.59E-13	141	1.66E-15	-4.29	4.55E-71	1.36E-67	7	0.00E+00	0.00E+00	-4.22	17.5	165.5
ENSRNOG00000031033	Mt-nd2	-0.90	1.46E-12	163	1.76E-14	-3.30	3.29E-48	4.29E-45	16	1.00E+00	8.99E-01	-3.22	20845.5	21024.5
ENSRNOG00000034234	Mt-cox1	-0.86	1.02E-11	190	1.42E-13	-3.19	3.11E-40	2.32E-37	28	1.00E+00	8.99E-01	-3.11	20845.5	21063.5
ENSRNOG00000030371	Mt-cox2	-0.70	1.53E-09	261	2.95E-11	-4.06	8.23E-47	1.01E-43	17	1.00E+00	8.99E-01	-3.98	20845.5	21123.5
ENSRNOG00000030700	Mt-cox3	-0.62	2.97E-08	330	7.24E-10	-4.58	1.02E-45	1.12E-42	19	1.00E+00	8.99E-01	-4.51	20845.5	21194.5
ENSRNOG00000033299	Mt-atp8	-0.60	5.47E-08	348	1.40E-09	-4.53	6.64E-51	9.22E-48	15	0.00E+00	0.00E+00	-4.45	17.5	380.5
ENSRNOG00000031979	Mt-atp6	-0.58	1.28E-07	378	3.57E-09	-4.57	9.50E-44	7.92E-41	25	1.00E+00	8.99E-01	-4.50	20845.5	21248.5
ENSRNOG00000033615	Mt-nd3	-0.39	9.20E-05	760	5.15E-06	-4.51	1.92E-32	9.33E-30	43	0.00E+00	0.00E+00	-4.45	17.5	820.5
ENSRNOG00000032609	Mt_tRNA	0.06	7.83E-01	15677	6.31E-01	0.21	4.88E-01	6.87E-01	14809	9.95E-01	8.66E-01	0.29	15521	46007

Neuronal Cultures

Ensemble Gene ID	Gene ID	log2Fold change (DESeq2)	pAdj (DESeq2)	rank (DESeq)	pValue (DESeq2)	log2Fold change (edgeR)	pValue (edgeR)	pAdj (edgeR)	rank (edgeR)	1-Likelihood (baySeq)	AdjLikelihood (baySeq)	log2Fold change (raw)	rank (baySeq)	Sum of ranks
ENSRNOG00000029042	**Mt-nd6**	**-5.63**	**0.00E+00**	**8**	0.00E+00	-6.70	0.00E+00	0.00E+00	9	0.00E+00	0.00E+00	-6.66	161	178
ENSRNOG00000029707	**Mt-nd4**	**-5.65**	**0.00E+00**	**8**	0.00E+00	-6.83	0.00E+00	0.00E+00	9	0.00E+00	0.00E+00	-6.78	161	178
ENSRNOG00000029971	**Mt-nd5**	**-5.70**	**0.00E+00**	**8**	0.00E+00	-6.75	0.00E+00	0.00E+00	9	0.00E+00	0.00E+00	-6.71	161	178
ENSRNOG00000030371	**Mt-cox2**	**-6.08**	**0.00E+00**	**8**	0.00E+00	-6.82	0.00E+00	0.00E+00	9	0.00E+00	0.00E+00	-6.77	161	178
ENSRNOG00000030478	**Mt_rRNA**	**-3.80**	**0.00E+00**	**8**	0.00E+00	-3.81	0.00E+00	0.00E+00	9	0.00E+00	0.00E+00	-3.77	161	178
ENSRNOG00000030644	**Mt-nd1**	**-5.66**	**0.00E+00**	**8**	0.00E+00	-6.12	0.00E+00	0.00E+00	9	0.00E+00	0.00E+00	-6.08	161	178
ENSRNOG00000030700	**Mt-cox3**	**-5.76**	**0.00E+00**	**8**	0.00E+00	-7.04	0.00E+00	0.00E+00	9	0.00E+00	0.00E+00	-7.00	161	178
ENSRNOG00000031033	**Mt-nd2**	**-6.23**	**0.00E+00**	**8**	0.00E+00	-6.64	0.00E+00	0.00E+00	9	0.00E+00	0.00E+00	-6.60	161	178
ENSRNOG00000031053	**Mt-nd4l**	**-5.56**	**0.00E+00**	**8**	0.00E+00	-6.87	0.00E+00	0.00E+00	9	0.00E+00	0.00E+00	-6.84	161	178
ENSRNOG00000031766	**Mt-cyb**	**-6.06**	**0.00E+00**	**8**	0.00E+00	-7.15	0.00E+00	0.00E+00	9	0.00E+00	0.00E+00	-7.11	161	178
ENSRNOG00000031979	**Mt-atp6**	**-5.91**	**0.00E+00**	**8**	0.00E+00	-7.03	0.00E+00	0.00E+00	9	0.00E+00	0.00E+00	-6.98	161	178
ENSRNOG00000033299	**Mt-atp8**	**-6.14**	**0.00E+00**	**8**	0.00E+00	-7.08	0.00E+00	0.00E+00	9	0.00E+00	0.00E+00	-7.04	161	178
ENSRNOG00000034234	**Mt-cox1**	**-6.15**	**0.00E+00**	**8**	0.00E+00	-6.42	0.00E+00	0.00E+00	9	0.00E+00	0.00E+00	-6.38	161	178
ENSRNOG00000043866	**Mt_rRNA**	**-4.32**	**0.00E+00**	**8**	0.00E+00	-4.32	0.00E+00	0.00E+00	9	0.00E+00	0.00E+00	-4.28	161	178
ENSRNOG00000018824	Slc7a5	**1.63**	**0.00E+00**	**8**	0.00E+00	1.71	8.36E-301	9.24E-298	19	0.00E+00	0.00E+00	1.75	161	188
ENSRNOG00000004133	Slc7a3	**2.16**	**3.14E-276**	**16**	3.68E-279	2.29	0.00E+00	0.00E+00	9	0.00E+00	0.00E+00	2.33	161	186
ENSRNOG00000007546	Asns	**1.36**	**1.22E-231**	**17**	1.52E-234	1.44	1.45E-313	1.70E-310	18	0.00E+00	0.00E+00	1.48	161	196
ENSRNOG00000014573	**Ckmt1b**	**-2.39**	**1.10E-224**	**18**	1.45E-227	-2.43	5.32E-292	5.59E-289	20	0.00E+00	0.00E+00	-2.39	161	199
ENSRNOG00000018487	Slc3a2	**1.07**	**1.81E-214**	**19**	2.52E-217	1.15	8.34E-218	7.97E-215	22	0.00E+00	0.00E+00	1.19	161	202
ENSRNOG00000020060	Atf5	**1.52**	**3.27E-198**	**20**	4.80E-201	1.61	3.36E-283	3.36E-280	21	0.00E+00	0.00E+00	1.65	161	202
**Other mitochondrial RNA**														
ENSRNOG00000029145	Mt_tRNA	**-4.52**	**4.25E-193**	**22**	6.86E-196	-6.59	0.00E+00	0.00E+00	9	0.00E+00	0.00E+00	-6.57	161	192
ENSRNOG00000033615	Mt-nd3	**-4.55**	**7.86E-159**	**27**	1.56E-161	-6.91	0.00E+00	0.00E+00	9	0.00E+00	0.00E+00	-6.87	161	197

Astrocyte Cultures

Ensemble Gene ID	Gene ID	log2Fold change (DESeq2)	pAdj (DESeq2)	rank (DESeq)	pValue (DESeq2)	log2Fold change (edgeR)	pValue (edgeR)	pAdj (edgeR)	rank (edgeR)	1-Likelihood (baySeq)	AdjLikelihood (baySeq)	log2Fold change (raw)	rank (baySeq)	Sum of ranks
ENSRNOG00000028108	Cytl1	**-1.97**	**1.04E-135**	**1**	7.29E-140	-2.07	6.26E-148	1.28E-143	1	7.11E-15	6.46E-16	-2.07	11.5	13.5
ENSRNOG00000007546	Asns	**1.73**	**1.53E-129**	**2**	2.14E-133	1.77	7.27E-128	4.94E-124	3	2.84E-06	3.97E-07	1.79	130	135
ENSRNOG00000020060	Atf5	**1.46**	**1.92E-113**	**3**	4.03E-117	1.48	7.58E-112	3.86E-108	4	7.70E-06	1.10E-06	1.49	153	160
ENSRNOG00000005695	Mgp	**-1.46**	**4.92E-108**	**4**	1.38E-111	-1.52	6.58E-128	4.94E-124	2	9.95E-14	1.23E-14	-1.51	15	21
ENSRNOG00000018414	Csf1r	**-1.71**	**1.88E-104**	**5**	6.60E-108	-1.80	1.70E-110	6.92E-107	5	2.11E-12	5.06E-13	-1.80	26	36
ENSRNOG00000025691	Pla2g7	**1.97**	**6.41E-101**	**6**	2.69E-104	2.07	7.79E-108	2.65E-104	6	0.00E+00	0.00E+00	2.07	5.5	17.5
ENSRNOG00000002393	Eprs	**1.29**	**3.49E-91**	**7**	1.71E-94	1.30	4.03E-73	6.31E-70	13	4.72E-01	1.31E-01	1.31	2444	2464
ENSRNOG00000019525	Hspa9	**1.16**	**1.38E-90**	**8**	7.76E-94	1.17	4.73E-78	8.76E-75	11	3.16E-03	6.51E-04	1.18	543	562
ENSRNOG00000017333	Syt4	**2.72**	**1.26E-76**	**9**	7.96E-80	3.24	9.07E-74	1.54E-70	12	1.39E-08	1.19E-09	3.25	63	84
ENSRNOG00000010645	Lgals3	**1.30**	**1.62E-73**	**10**	1.13E-76	1.32	6.18E-60	5.47E-57	23	9.96E-01	8.28E-01	1.33	17949	17982

ENSRNOG00000028648	Olig1	**-1.08**	**5.01E-70**	**11**	3.86E-73	-1.13	3.17E-64	3.40E-61	19	6.88E-06	9.30E-07	-1.12	149	179
ENSRNOG00000010210	Slc7a11	**1.44**	**9.57E-70**	**12**	8.05E-73	1.48	6.99E-85	1.78E-81	8	0.00E+00	0.00E+00	1.49	5.5	25.5
ENSRNOG00000008106	Shmt2	**1.19**	**4.28E-69**	**13**	3.90E-72	1.20	3.45E-70	5.02E-67	14	2.80E-06	3.59E-07	1.22	128	155
ENSRNOG00000027491	Vldlr	**1.27**	**1.47E-68**	**14**	1.44E-71	1.30	5.44E-80	1.11E-76	10	6.22E-11	7.07E-12	1.31	37	61
ENSRNOG00000012582	Eif4ebp1	**1.38**	**1.90E-61**	**15**	2.00E-64	1.42	9.64E-66	1.09E-62	18	1.32E-08	9.86E-10	1.44	62	95
ENSRNOG00000050190	Eng	**-1.69**	**3.12E-61**	**16**	3.50E-64	-1.83	1.50E-80	3.39E-77	9	7.86E-12	1.24E-12	-1.83	29	54
ENSRNOG00000021269	Chgb	**1.04**	**2.57E-60**	**17**	3.06E-63	1.04	4.64E-67	5.57E-64	17	1.68E-07	2.08E-08	1.06	87	121
ENSRNOG00000027468	Slc6a15	**1.70**	**4.77E-60**	**18**	6.01E-63	1.82	3.24E-63	3.15E-60	21	0.00E+00	0.00E+00	1.83	5.5	44.5
ENSRNOG00000005669	Car8	**1.96**	**8.71E-59**	**19**	1.16E-61	2.16	6.68E-64	6.81E-61	20	2.67E-11	3.58E-12	2.18	34	73
ENSRNOG00000017484	Gja5	**-1.24**	**4.29E-58**	**20**	6.01E-61	-1.31	1.72E-55	1.17E-52	30	6.50E-07	9.11E-08	-1.31	106	156
**Other mitochondrial RNA**														
ENSRNOG00000030478	Mt_rRNA	**2.20**	**3.85E-29**	**105**	2.83E-31	3.54	4.24E-86	1.24E-82	7	1.00E+00	8.48E-01	3.55	20370	20482
ENSRNOG00000029042	Mt-nd6	**-1.38**	**1.24E-13**	**357**	3.10E-15	-1.92	3.34E-45	1.15E-42	59	4.27E-02	9.12E-03	-1.90	1095	1511
ENSRNOG00000031766	Mt-cyb	**-1.31**	**3.14E-13**	**371**	8.17E-15	-1.76	1.54E-38	3.54E-36	89	2.33E-01	5.44E-02	-1.74	1818	2278
ENSRNOG00000029971	Mt-nd5	**-1.22**	**4.06E-13**	**378**	1.08E-14	-1.56	1.14E-37	2.44E-35	95	1.11E-04	1.93E-05	-1.54	243	716
ENSRNOG00000030700	Mt-cox3	**-1.14**	**2.53E-12**	**404**	7.17E-14	-1.42	2.37E-32	4.02E-30	120	2.28E-01	5.33E-02	-1.39	1807	2331
ENSRNOG00000033615	Mt-nd3	**-0.95**	**2.24E-08**	**692**	1.09E-09	-1.20	1.47E-25	1.58E-23	190	9.99E-01	8.44E-01	-1.18	19876	20758
ENSRNOG00000043866	Mt_rRNA	**1.04**	**2.62E-07**	**824**	1.52E-08	1.51	6.09E-24	5.91E-22	210	9.90E-01	7.89E-01	1.51	14556	15590
ENSRNOG00000032609	Mt_tRNA	**1.08**	**3.18E-07**	**838**	1.87E-08	1.77	7.96E-11	2.38E-09	680	2.06E-03	4.15E-04	1.78	485	2003
ENSRNOG00000029707	Mt-nd4	**-1.02**	**4.69E-07**	**863**	2.83E-08	-1.56	3.84E-22	3.42E-20	229	7.34E-01	2.66E-01	-1.53	3384	4476
ENSRNOG00000031053	Mt-nd4l	**-0.99**	**2.09E-06**	**983**	1.44E-07	-1.56	4.78E-22	4.20E-20	232	7.55E-03	1.63E-03	-1.53	693	1908
ENSRNOG00000030371	Mt-cox2	**-0.76**	**8.75E-05**	**1458**	8.94E-06	-1.04	3.39E-14	1.50E-12	460	9.65E-01	6.60E-01	-1.02	8664	10582
ENSRNOG00000031979	Mt-atp6	**-0.78**	**1.12E-04**	**1503**	1.18E-05	-1.12	3.24E-14	1.44E-12	458	9.48E-01	5.65E-01	-1.09	6565	8526
ENSRNOG00000033299	Mt-atp8	**-0.63**	**6.38E-03**	**2681**	1.20E-03	-1.16	2.03E-09	5.06E-08	817	9.99E-01	8.45E-01	-1.13	20024	23522
ENSRNOG00000029145	Mt_tRNA	**0.29**	**1.15E-01**	**5057**	4.00E-02	0.32	4.05E-02	1.37E-01	6039	9.08E-01	4.65E-01	0.34	5158	16254
ENSRNOG00000034234	Mt-cox1	**-0.32**	**1.22E-01**	**5149**	4.33E-02	-0.43	8.00E-04	5.75E-03	2837	1.00E+00	8.47E-01	-0.41	20325	28311
ENSRNOG00000030644	Mt-nd1	**-0.04**	**9.06E-01**	**18216**	8.28E-01	-0.08	6.34E-01	7.76E-01	16660	1.00E+00	8.47E-01	-0.05	20316	55192
ENSRNOG00000031033	Mt-nd2	**0.03**	**9.14E-01**	**18410**	8.43E-01	0.03	8.61E-01	9.30E-01	18865	1.00E+00	8.47E-01	0.05	20327	57602
