# Supplementary material for: Mitochondrial DNA depletion by ethidium bromide decreases neuronal mitochondrial creatine kinase: Implications for striatal energy metabolism
Source: PLoS One. 2017 Dec 29;12(12):e0190456. doi: 10.1371/journal.pone.0190456 (PMC5747477; doi:10.1371/journal.pone.0190456)
Supplement: S4 Table — The 2000, 1000, and 500 genes most significantly regulated by EtBr treatment in all three culture conditions were assessed for directionality of regulation. Across conditions, significantly regulated genes were approximately evenly distributed between up- and downregulated groups. (PDF) [file pone.0190456.s009.pdf]

S4 Table. Distribution of the most significantly up- and downregulated genes analyzed with MultiRankSeq.

Cell type	Total	# upregulated	# downregulated	% upregulated	% downregulated
**NECos**	2000 most significant	1034	966	52%	48%
**NECos**	1000 most significant	530	470	53%	47%
**NECos**	500 most significant	275	225	55%	45%
**Neurons**	2000 most significant	981	1019	49%	51%
**Neurons**	1000 most significant	464	536	46%	54%
**Neurons**	500 most significant	199	301	40%	60%
**Astrocytes**	2000 most significant	1039	961	52%	48%
**Astrocytes**	1000 most significant	531	469	53%	47%
**Astrocytes**	500 most significant	264	236	53%	47%
