# Supplementary material for: Mitochondrial DNA depletion by ethidium bromide decreases neuronal mitochondrial creatine kinase: Implications for striatal energy metabolism
Source: PLoS One. 2017 Dec 29;12(12):e0190456. doi: 10.1371/journal.pone.0190456 (PMC5747477; doi:10.1371/journal.pone.0190456)
Supplement: S5 Table — NIH DAVID was used for pathway analysis of RNASeq data, with focus on the UniProt and KEGG databases, which have in-depth characterization of gene groups [69]. The 500 strongest downregulated and 500 strongest upregulated genes after EtBr treatment (50 ng/ml, 4 days), ranked with DESeq2, which together made up less than 5% of all genes, were used in separate analyses. Category = Original database Term = Enriched terms associated with input gene list Count = Genes involved in term % = involved genes/total genes PValue = modified Fisher’s exact p-value Bonferroni = Bonferroni-corrected p-value Benjamini = Benjamini-Hochberg corrected p-value FDR = False discovery rate (PDF) [file pone.0190456.s010.pdf]

S5 Table. NIH DAVID analysis of RNASeq data.

(a) NeCo

Enriched terms in upregulated genes

Category	Term	Count	%	PValue	Bonferroni	Benjamini	FDR
KEGG_PATHWAY	Ribosome	42	8.7	8.06E-25	1.81E-22	1.81E-22	1.03E-21
UP_KEYWORDS	Ribosomal protein	43	8.9	7.92E-24	2.19E-21	7.28E-22	1.04E-20
UP_KEYWORDS	Protein biosynthesis	21	4.4	1.10E-12	3.05E-10	5.08E-11	1.46E-09
UP_KEYWORDS	Ubl conjugation	53	11.0	1.03E-11	2.85E-09	4.08E-10	1.36E-08
UP_KEYWORDS	Neurogenesis	18	3.7	4.73E-08	1.30E-05	1.63E-06	6.23E-05
UP_KEYWORDS	Aminoacyl-tRNA synthetase	10	2.1	2.46E-07	6.78E-05	7.54E-06	3.24E-04
KEGG_PATHWAY	Aminoacyl-tRNA biosynthesis	11	2.3	7.17E-05	1.60E-02	8.04E-03	9.16E-02
UP_KEYWORDS	ATP-binding	44	9.1	1.27E-04	3.45E-02	2.06E-03	1.67E-01
KEGG_PATHWAY	Central carbon metabolism in cancer	10	2.1	2.09E-04	4.60E-02	1.17E-02	2.67E-01
KEGG_PATHWAY	Glycolysis / Gluconeogenesis	10	2.1	4.17E-04	8.95E-02	1.86E-02	5.31E-01
UP_KEYWORDS	Amino-acid transport	6	1.2	4.78E-04	1.24E-01	6.57E-03	6.28E-01
UP_KEYWORDS	Sodium/potassium transport	4	0.8	6.24E-04	1.58E-01	8.17E-03	8.20E-01
UP_KEYWORDS	Glycolysis	6	1.2	1.17E-03	2.75E-01	1.33E-02	1.53E+00
KEGG_PATHWAY	Glucagon signaling pathway	11	2.3	1.51E-03	2.88E-01	5.49E-02	1.91E+00
KEGG_PATHWAY	Butanoate metabolism	6	1.2	1.89E-03	3.47E-01	5.91E-02	2.39E+00
KEGG_PATHWAY	Carbon metabolism	12	2.5	1.93E-03	3.53E-01	5.30E-02	2.44E+00
KEGG_PATHWAY	GABAergic synapse	10	2.1	2.03E-03	3.67E-01	4.47E-02	2.56E+00
INTERPRO	Immunoglobulin I-set	11	2.3	2.25E-03	9.22E-01	3.46E-01	3.55E+00
KEGG_PATHWAY	Biosynthesis of amino acids	9	1.9	5.51E-03	7.11E-01	9.11E-02	6.81E+00
KEGG_PATHWAY	Glycine, serine and threonine metabolism	6	1.2	8.28E-03	8.46E-01	1.04E-01	1.01E+01

Enriched terms in downregulated genes

Category	Term	Count	%	PValue	Bonferroni	Benjamini	FDR
UP_KEYWORDS	Respiratory chain	10	2.1	4.93E-07	1.38E-04	6.88E-05	6.52E-04
UP_KEYWORDS	Electron transport	11	2.3	5.94E-06	1.66E-03	5.52E-04	7.85E-03
KEGG_PATHWAY	Oxidative phosphorylation	15	3.1	7.46E-06	1.56E-03	1.56E-03	9.41E-03
UP_KEYWORDS	Ubiquinone	8	1.7	8.61E-06	2.40E-03	6.00E-04	1.14E-02
UP_KEYWORDS	Mitochondrion	30	6.2	8.23E-04	2.05E-01	2.83E-02	1.08E+00
UP_KEYWORDS	Lipoprotein	22	4.6	4.35E-03	7.03E-01	1.14E-01	5.59E+00
UP_KEYWORDS	Mitochondrion inner membrane	11	2.3	8.34E-03	9.03E-01	1.44E-01	1.05E+01

(b) Neurons

Enriched terms in upregulated genes

Category	Term	Count	%	PValue	Bonferroni	Benjamini	FDR
KEGG_PATHWAY	Ribosome	38	7.8	1.07E-23	1.85E-21	1.85E-21	1.31E-20
UP_KEYWORDS	Ribosomal protein	38	7.8	4.40E-19	1.04E-16	5.18E-17	5.67E-16
UP_KEYWORDS	Aminoacyl-tRNA synthetase	12	2.5	1.19E-09	2.81E-07	5.61E-08	1.54E-06

UP_KEYWORDS	Protein biosynthesis	16	3.3	4.97E-08	1.17E-05	1.95E-06	6.39E-05
UP_KEYWORDS	Ubl conjugation	45	9.2	8.44E-08	1.98E-05	2.48E-06	1.09E-04
KEGG_PATHWAY	Aminoacyl-tRNA biosynthesis	13	2.7	2.62E-07	4.53E-05	2.27E-05	3.21E-04
UP_KEYWORDS	Amino-acid transport	7	1.4	4.59E-05	1.07E-02	8.99E-04	5.91E-02
UP_KEYWORDS	Transcription	40	8.2	8.97E-05	2.09E-02	1.62E-03	1.15E-01
UP_KEYWORDS	Repressor	15	3.1	4.18E-04	9.36E-02	6.12E-03	5.37E-01
UP_KEYWORDS	Zinc-finger	36	7.4	7.95E-04	1.71E-01	1.09E-02	1.02E+00

Enriched terms in downregulated genes

Category	Term	Count	%	PValue	Bonferroni	Benjamini	FDR
UP_KEYWORDS	Steroid biosynthesis	16	3.3	7.96E-17	3.14E-14	7.88E-15	1.44E-13
UP_KEYWORDS	Sterol biosynthesis	14	2.9	1.68E-16	6.28E-14	1.25E-14	2.89E-13
UP_KEYWORDS	Cholesterol biosynthesis	12	2.5	9.04E-15	2.54E-12	3.18E-13	1.19E-11
UP_KEYWORDS	Lipid metabolism	35	7.3	3.04E-14	8.61E-12	9.57E-13	4.03E-11
UP_KEYWORDS	Oxidoreductase	43	9.0	3.00E-13	8.48E-11	7.71E-12	3.97E-10
UP_KEYWORDS	Mitochondrion	48	10.0	7.16E-12	2.03E-09	1.45E-10	9.48E-09
KEGG_PATHWAY	Carbon metabolism	21	4.4	1.91E-09	4.48E-07	8.96E-08	2.45E-06
KEGG_PATHWAY	Glycolysis / Gluconeogenesis	15	3.1	4.55E-08	1.07E-05	1.78E-06	5.86E-05
UP_KEYWORDS	Glycolysis	10	2.1	6.78E-08	1.92E-05	1.07E-06	8.98E-05
UP_KEYWORDS	Electron transport	13	2.7	8.17E-08	2.31E-05	1.22E-06	1.08E-04
UP_KEYWORDS	Mitochondrion inner membrane	19	4.0	1.46E-07	4.12E-05	2.06E-06	1.93E-04
KEGG_PATHWAY	Oxidative phosphorylation	20	4.2	2.10E-07	4.94E-05	7.06E-06	2.70E-04
KEGG_PATHWAY	Biosynthesis of amino acids	15	3.1	4.25E-07	9.98E-05	1.25E-05	5.47E-04
UP_KEYWORDS	Respiratory chain	10	2.1	4.60E-07	1.30E-04	6.20E-06	6.10E-04
KEGG_PATHWAY	Pyruvate metabolism	10	2.1	4.39E-06	1.03E-03	1.03E-04	5.65E-03
KEGG_PATHWAY	GABAergic synapse	14	2.9	4.55E-06	1.07E-03	9.73E-05	5.86E-03
KEGG_PATHWAY	Citrate cycle (TCA cycle)	8	1.7	6.40E-05	1.49E-02	1.07E-03	8.22E-02
UP_KEYWORDS	Fatty acid metabolism	10	2.1	5.67E-04	1.48E-01	3.64E-03	7.48E-01
INTERPRO	Phosphofructokinase, conserved site	3	0.6	1.66E-03	8.20E-01	2.91E-01	2.60E+00
KEGG_PATHWAY	Butanoate metabolism	6	1.3	1.96E-03	3.69E-01	2.07E-02	2.49E+00
KEGG_PATHWAY	Pentose phosphate pathway	6	1.3	2.30E-03	4.19E-01	2.33E-02	2.92E+00
KEGG_PATHWAY	Alanine, aspartate and glutamate metabolism	6	1.3	5.37E-03	7.18E-01	4.75E-02	6.69E+00

(c) Astrocytes

Enriched terms in upregulated genes

Category	Term	Count	%	PValue	Bonferroni	Benjamini	FDR
UP_KEYWORDS	Aminoacyl-tRNA synthetase	13	2.7	5.92E-11	1.77E-08	8.85E-09	7.90E-08
UP_KEYWORDS	ATP-binding	55	11.4	1.39E-08	4.16E-06	6.93E-07	1.86E-05
KEGG_PATHWAY	Aminoacyl-tRNA biosynthesis	14	2.9	9.26E-08	2.13E-05	2.13E-05	1.19E-04
UP_KEYWORDS	Amino-acid biosynthesis	8	1.7	5.79E-07	1.73E-04	1.73E-05	7.73E-04
UP_KEYWORDS	Amino-acid transport	8	1.7	3.27E-06	9.77E-04	8.89E-05	4.37E-03
KEGG_PATHWAY	Biosynthesis of amino acids	13	2.7	5.53E-06	1.27E-03	4.24E-04	7.09E-03

KEGG_PATHWAY	Central carbon metabolism in cancer	10	2.1	1.05E-04	2.39E-02	4.83E-03	1.35E-01
INTERPRO	L-type amino acid transporter	3	0.6	1.71E-03	8.41E-01	1.03E-01	2.69E+00
KEGG_PATHWAY	Carbon metabolism	11	2.3	3.21E-03	5.22E-01	7.88E-02	4.03E+00
UP_KEYWORDS	Lipid metabolism	16	3.3	5.05E-03	7.80E-01	4.92E-02	6.53E+00
KEGG_PATHWAY	Glycine, serine and threonine metabolism	6	1.2	5.68E-03	7.31E-01	1.12E-01	7.05E+00

Enriched terms in downregulated genes

Category	Term	Count	%	PValue	Bonferroni	Benjamini	FDR
UP_KEYWORDS	Immunity	27	5.5	1.18E-11	3.28E-09	3.28E-09	1.55E-08
KEGG_PATHWAY	DNA replication	14	2.9	6.47E-11	1.46E-08	1.46E-08	8.27E-08
KEGG_PATHWAY	B cell receptor signaling pathway	18	3.7	7.24E-11	1.64E-08	8.18E-09	9.26E-08
UP_KEYWORDS	Inflammatory response	14	2.9	3.82E-08	1.07E-05	2.13E-06	5.05E-05
UP_KEYWORDS	ATP-binding	44	9.0	1.59E-04	4.35E-02	4.93E-03	2.10E-01
UP_KEYWORDS	Lipid biosynthesis	11	2.3	3.58E-04	9.51E-02	9.95E-03	4.72E-01
UP_KEYWORDS	Ubiquinone	6	1.2	9.27E-04	2.28E-01	1.97E-02	1.22E+00
UP_KEYWORDS	Respiratory chain	6	1.2	3.11E-03	5.81E-01	4.47E-02	4.03E+00
UP_KEYWORDS	Lipid metabolism	16	3.3	5.65E-03	7.94E-01	6.65E-02	7.22E+00
KEGG_PATHWAY	cAMP signaling pathway	15	3.1	6.21E-03	7.55E-01	5.27E-02	7.66E+00
